# Recent Advances in Pd-Based Membranes for Membrane Reactors

**DOI:** 10.3390/molecules22010051

**Published:** 2017-01-01

**Authors:** Alba Arratibel Plazaola, David Alfredo Pacheco Tanaka, Martin Van Sint Annaland, Fausto Gallucci

**Affiliations:** 1 Chemical Process Intensification, Department of Chemical Engineering and Chemistry, Eindhoven University of Technology, P.O. Box 513, 5612 AZ Eindhoven, The Netherlands; A.Arratibel.Plazaola@tue.nl (A.A.P.); M.v.SintAnnaland@tue.nl (M.V.S.A.); 2 Tecnalia, Mikeletegi Pasealekua 2, 20009 Donostia-San Sebastian, Spain; alfredo.pacheco@tecnalia.com

**Keywords:** membranes, membrane reactor, pore-filled, palladium membranes, inorganic membranes

## Abstract

Palladium-based membranes for hydrogen separation have been studied by several research groups during the last 40 years. Much effort has been dedicated to improving the hydrogen flux of these membranes employing different alloys, supports, deposition/production techniques, etc. High flux and cheap membranes, yet stable at different operating conditions are required for their exploitation at industrial scale. The integration of membranes in multifunctional reactors (membrane reactors) poses additional demands on the membranes as interactions at different levels between the catalyst and the membrane surface can occur. Particularly, when employing the membranes in fluidized bed reactors, the selective layer should be resistant to or protected against erosion. In this review we will also describe a novel kind of membranes, the pore-filled type membranes prepared by Pacheco Tanaka and coworkers that represent a possible solution to integrate thin selective membranes into membrane reactors while protecting the selective layer. This work is focused on recent advances on metallic supports, materials used as an intermetallic diffusion layer when metallic supports are used and the most recent advances on Pd-based composite membranes. Particular attention is paid to improvements on sulfur resistance of Pd based membranes, resistance to hydrogen embrittlement and stability at high temperature.

## 1. Introduction

When compared with pressure swing adsorption (PSA) and cryogenic distillation, hydrogen permeable Pd-based membranes are a very promising alternative for the separation of pure hydrogen at small- and medium-scales, while concerns about the Pd availability could hamper the exploitation of this kind of membranes at very large industrial scales, such as pre-combustion capture [1,2,3,4,5]. Different combinations of supports, membrane materials and alloys have been investigated with the aim of developing high-flux membranes showing long-term stability at industrially relevant conditions. The early investigations were based on self-supported thick membranes which presented a very high selectivity (required for niche applications such as nuclear), but low permeation rates and extremely high costs. As the U.S. Department Of Energy (US DOE) targets for palladium membranes (used as reference in many countries, including Europe) are getting more demanding every 5 years [6], more efforts have been made toward the development of supported thin films due to the improvements in mechanical stability while maintaining high hydrogen permeation rates. Several techniques are used for the preparation of (dense) supported palladium membranes (electroless plating, electro-deposition, physical vapor deposition and chemical vapour deposition), which were well described by Yun and Oyama [7]. Membrane preparation methods were also reported by Paglieri and Way in 2002, who described their application in a membrane reactor, fuel cell, hydrogen isotopes separation and novel applications such as quantification of produced hydrogen by microbes, among others [8].

Before separation, hydrogen is produced mainly by steam reforming (SR) or autothermal (ATR) reactions of different feedstocks, such as methane, higher hydrocarbons, methanol and ethanol; the produced hydrogen can be separated downstream from the exhaust gas stream using hydrogen perm-selective membranes. More interestingly, the separation can be integrated in the reactor (in a membrane reactor—MR) such that the in-situ hydrogen separation will also positively affect the conversion and selectivity of the often equilibrium limited reactions (shift effect according to Le Châtelier’s principle).

When using a membrane reactor, the practical configuration can be either in a packed (fixed) bed or as a fluidized bed, where the catalyst is free to move around the membranes. Micro-structured membrane reactors have also been investigated in view of their good heat and mass transfer characteristics [9,10,11,12,13], however, the exploitation of this kind of configuration is hampered by the very active catalytic activity required [10], the extremely high surface area to catalyst volume ratio (in the order 10^5^ m^2^/m^3^ [13]) and by the challenging manifolding and difficulty of accessing damaged channels in a system where hundreds of channels would be required even for smallscale applications.

**Figure 1:**
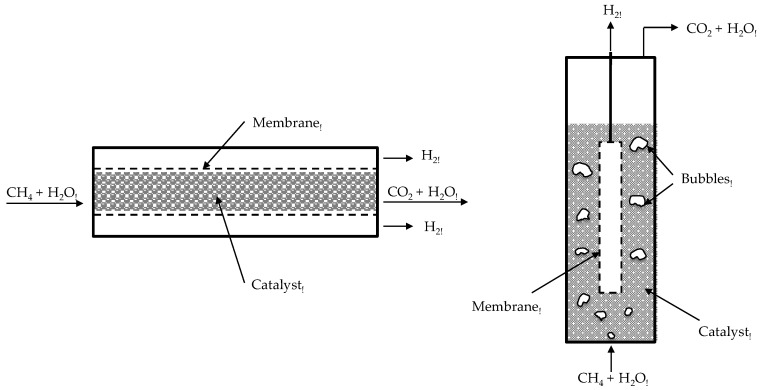
Schematic representation of packed-bed (**left**) and fluidized-bed (**right**) membrane reactors.

The advantages of packed-beds lie in its simplicity in construction and well established and validated models for its design and scale-up. Additionally, the catalyst is kept in a fixed position, thus any damage of the membranes due to erosion is circumvented while scratches on the thin membrane surface can only happen when loading and unloading the catalyst from the reactor. On the other hand, the main disadvantages of this kind of reactors are the unavoidable temperature gradients that the reactor (and thus the membrane) is experiencing in both endothermic (steam reforming like) or exothermic (autothermal reforming) reaction systems [14]. More importantly, as thinner membranes are produced with much higher fluxes, the concentration polarization (or better the bed-to-wall mass transfer limitation) prevailing in packed beds will be extremely detrimental for the hydrogen recovery and thus for the overall performance of the membrane reactor [14,15]. These limitations affect fluidized beds to a lesser extent, since they can be operated at virtually uniform temperature and reduced mass transfer limitations. However, much more attention should be paid to possible damages of membranes by erosion. This was already reported in a previous review by Gallucci et al. [16], where the authors showed and discussed in detail different reactor configurations, such as packed-bed (Figure 1 left) and fluidized-bed (Figure 1 right) membrane reactors as well as other concepts. 

A recent paper by Fernandez et al. shows that the integration of membranes in membrane reactors can be also hampered by chemical interaction between the membrane itself and (some component of) the catalyst used in the reactor [17]. For instance they showed that by using a catalyst bed containing TiO_2_ a strong interaction between this component and the membrane surface made the permeation flux decrease by a factor of 14 within a few h of experiments under fluidized conditions. Other tests with the same material and powder in a packed bed configuration showed the same effect with an ever higher decrease up to 25 times lower flux (unpublished tests).

Compared with our previous review [16], the present paper reports novel trends by collecting the latest papers published on Pd-membranes and considering the most relevant information for membrane reactor applications. This review is outlined as follows: First, different membrane reactor concepts for hydrogen production and separation from different feedstocks, viz. methane, methanol and ethanol, are reviewed, followed by a discussion of novel concepts to include CO_2_ capture. Subsequently, the characteristics and properties of the membranes are reviewed, focussing on: (a) effect of the support; (b) resistance of the membrane to embrittlement and poisoning with sulfur (by using palladium alloys); (c) resistance to damage caused by interactions with the catalyst by using cermet or “pore filled” membranes.

## 2. Membrane Reactors for Hydrogen Production and Separation

Integration of hydrogen perm-selective membranes into a catalytic reactor where hydrogen is produced by steam reforming or authothermal reforming of different feedstocks (methane, methanol, ethanol among other hydrocarbons), allows elimination of hydrogen purification units downstream of the reformer together with a reduction of total reactor volume [18]. Another positive aspect of using the membrane reactor concept is based on total capital cost reduction since milder operating conditions are required to achieve the same conversions of conventional systems due to hydrogen extraction through the membranes resulting in shifting of the reaction equilibria towards the products [19,20,21].

In the review presented by Gallucci et al. [16] the authors reported different configurations of membrane reactors (viz. packed-bed, fluidized-bed and micro-reactor) and the most recent advances obtained in the past 10 years. Since then a number of new works have been published focused on experimental studies for the combination of supported Pd-based membranes in membrane reactors for in situ hydrogen production and recovery. On the other hand, many more theoretical works were reported to confirm the superior performance of membrane reactors (in any configuration) compared with conventional systems [22,23,24,25,26,27,28,29,30,31]. 

As already reported above, the summary of these results show that packed bed membrane reactors (PBMR) are affected by severe mass and heat transfer limitations that result in reduced membrane fluxes (mass transfer limitations) and difficulties in maintaining a uniform temperature close to the membranes (heat transfer limitations) that could also eventually damage the membranes (due to hot spots in case of exothermic reactions). On the other hand, fluidized-bed membrane-reactors (FBMR), have much better heat and mass transfer rates [32], so that the membranes always experience a uniform temperature and the highest hydrogen partial pressure, thus maximizing both membrane flux and lifetime. Additionally, smaller particles can be used in fluidized beds because of the limited pressure drop, and thus any possible internal mass transfer limitation is circumvented. On the contrary, problems with membrane erosion and bubble-to-emulsion mass transfer limitations can affect the performance of the FBMR and the lifetime of the membranes [33]. An alternative to avoid direct contact between particles in constant motion with the membrane surface is to cover the surface with a porous layer with a porosity and pore size sufficiently large to avoid additional mass transfer limitations and small enough to avoid particles entering the pores. The porous structure can be a separate structure but physically attached to the membrane, or it can be part of the membrane itself. The second option can be achieved by using “pore-filled” membranes prepared with a very thin and porous ceramic layer covering the palladium deposited onto nanopores. Nevertheless, these kind of membranes were not yet tested inside a fluidized bed reactor. It should also be reminded that to protect the membrane the thin Pd layer cannot be deposited on the inner surface of a porous support (as one might immediately propose), because the large pressure difference used between the reaction zone (the fluidized bed) and the permeation zone (inside the tube) would result in fast delamination of the Pd layer with consequent loss in perm-selectivity.

Finally, Dang et al. [29,34] have also shown that in FBMRs, the application of extremely permeable membranes could lead to formation of densified zones close to the membrane walls in case mild fluidization of small particles is used. They concluded that it may preferable to work at higher fluidization velocities and with somewhat larger particles (in the order of few hundreds of microns), putting stronger targets on the membrane’s resistance against erosion.

### 2.1. Feedstock for H_2_ Production in MRs and Required Operating Conditions

As far as the feedstock for hydrogen production is concerned, most of the recent articles deal with methane and alcohols (methanol and ethanol) as fuel of the membrane reactor. Their conversion to H_2_, and selectivity to CO and CO_2_ depend on the operating conditions (temperature, pressure, reactor configuration and so on). Most of the reactions for hydrogen production (from either CO, methane, ethanol and methanol) are equilibrium limited reaction systems which are endothermic or exothermic depending on the H_2_O/C and O_2_/C ratios used. In Table 1 the most relevant reactions involving steam reforming for these three feedstocks and their enthalpies have been summarized.

The required temperatures for reforming of every feedstock are related to their endothermicity. In conventional reactors, methane and ethanol reforming require temperature above 800 °C and 600 °C respectively, while methanol reforming can be carried out at much lower temperatures of 250–300 °C [42].

These temperatures can be dramatically decreased when using membrane reactors as hydrogen recovery through Pd based membranes drives the reactions toward the products [43]. However, while having a clear energetic benefit, decreasing the reaction temperatures has also the negative effect of increasing the potential formation of carbon deposits on the catalysts. The catalysts for membrane reactors should therefore be more resistant against carbon formation. Alternatively excess of steam with typical molar ratios H_2_O/C of 3–5, can be used to avoid carbon formation [44]. Finally, to assure a long membrane lifetime the temperature of the reformer unit needs to be below the maximum operating temperature of membranes, which in case of Pd-based membranes is around 600 °C [45]. Indeed at this temperature the MR outperforms the conventional system, while the membrane area for hydrogen recovery is minimized [33].

In the following, the most recent results obtained with membrane reactors with integrated hydrogen perm-selective membranes for hydrogen production are presented by reforming of different feedstocks, viz. methane, methanol and ethanol. Table 2 summarizes the performance and operating conditions of the most recent studies reported in the last years, which are explained in more detail in the following sections. 

#### 2.1.1. Methane as Feedstock

Methane is the most frequently used (and consequently the most studied) light hydrocarbon for hydrogen production. As methane is a molecule that is difficult to activate, generally the reforming of methane is carried out at temperatures much higher than other hydrocarbons, particularly alcohols. When using natural gas as source of methane, the stream contains small amounts of H_2_S, which poison the surface of Pd-based membranes and may even lead to rupture of the thin membrane layer [46]. On the contrary, other feedstocks such as methanol and ethanol are much cleaner than natural gas, so that the reforming of such fuels is not detrimental for the Pd-membranes behaviour.

Larger conversion levels are achieved at lower temperatures (550–600 °C) in the MR, when compared with conventional methane steam reforming (800–900 °C). Additionally, the allowed decrease in temperature is also beneficial for the water gas shift (WGS) equilibrium, reducing the CO concentrations in the retentate stream [47].

Nickel-based catalysts represent the most effective option to achieve high conversions of methane at reasonable costs. Generally, these catalysts are supported on porous ceramics (i.e., γ-Al_2_O_3_ [48]). Others noble metals like rhodium were supported on ZrO_2_ for low temperature ATR of CH_4_ [10] as well as on YSZ for higher temperature ATR [49]. Noble metal catalysts are generally preferred over Ni-based catalyst for their higher reaction rates, higher stability and lower activity towards carbon formation. 

Problems associated to hot-spots in a packed-bed reactor were partially avoided using foam-supported catalysts as reported by Kyriakides et al. [50] in a two coaxial tubes MR. The temperature gradient was reduced from 100 °C to 25 °C as catalyst pellets were replaced by foam supported catalysts. Moreover, enhancement of methane conversion was achieved by replacing the catalyst due to better heat transfer: in fact when using commercial pellets up to 18.85% conversion at 1 bar and 500 °C average wall temperature (415 °C in the bed) was achieved, while the conversions observed for the foam-supported catalyst were up to 29.97% with the wall and bed temperature close to 500 °C.

The use of a catalytic foam in a membrane reactor was recently reported by Patrascu et al. [51]. A Pt_(3)_Ni_(10)_/CeO_2_ coated onto a SiC foam scaffold (high thermal conductivity) was used as a catalyst. The authors obtained 90% methane conversion and 80% of the produced hydrogen was recovered at 525 °C and 10 bar. This high conversion was achieved using 0.7 L∙min^−1^ of N_2_ sweep gas.

Integration of membranes into a packed-bed MR was reported by Matsuka et al. [52], where the authors compare three different types of self-supported membranes: 25 and 50-μm-thick Pd_77_Ag_23_, 100-μm-thick V and V_92_Ni_8_ layers coated on both sides with Pd by magnetron sputtering (1 μm). Experiments were performed in presence of 10 wt. % nickel catalysts supported on SiO_2_ while feeding a mixture of CH_4_, O_2_, H_2_O and N_2_. The best performance was observed for the Pd coated vanadium membrane, obtaining ~45% methane conversion at 400 °C while the permeation flux was 0.09 (mol∙m^−2^∙s^−1^). The membranes were exposed to Fe in a hydrogen environment at 500 °C, evidencing surface degradation of the PdAg membranes due to the formation of pinholes, while the presence of defects in V membranes was lower. EXD analyses on the cross sections showed traces of iron diffusion along the PdAg membrane. On the other hand, interestingly no Fe diffusion through the Pd/V/Pd membrane was detected, while intermetallic diffusion of palladium into vanadium through 10–15 μm in depth was observed.

Methane reforming in a packed bed membrane reactor was also performed by Silva et al. [56] using a 76.2 μm thick Pd-Ag membrane and a CH_4_/CO_2_ ratio of 2.85 at 600 °C resulting in 35% methane conversion, while 47% of the produced hydrogen was recovered. Larger methane conversions were obtained by Gil et al. [53] using a catalytic hollow fibre membrane reactor were Ni (25 wt. %)/SBA-15 catalyst was coated on the inside of an Al_2_O_3_ hollow fibre. A methane conversion of 53% was reached at 560 °C and 43% of the produced hydrogen was recovered through a 3.3 μm thick Pd layer deposited on top of the hollow fibre.

Pd membranes deposited by electroless plating (10.2 μm) on top of an oxidized porous stainless steel substrate produced by Sanz et al. [54] were evaluated in a WGS membrane reactor. Two configurations were tested employing commercial catalysts consisting of a Fe-Cr oxide: a conventional packed bed reactor and a membrane reactor. The membrane was tested in the presence of a methane reformate mixture consisting of 70% H_2_, 18% CO and 12% CO_2_. The presence of CO and CO_2_ decreased the permeated hydrogen flux due to carbon deposition and CO adsorption on the membrane surface [82,83]. The measured CO conversions for the membrane reactor at a transmembrane pressure difference of 3 bar were much higher than the obtained conversions in the packed-bed reactor in the temperature range of 350–400 °C, even though the recovered hydrogen through the membrane was less than 15% in the investigated temperature range and pressure difference of 2–3 bar. Goldbach et al. [84] tested, for 150 days, under WGS reformate mixture (53.9% H_2_, 28.1% H_2_O, 16.3% CO_2_, 1.1% CO and 0.4% CH_4_) a 2–4 μm thick Pd-membrane at 400 °C and transmembrane pressure difference of 1800–2000 kPa. The hydrogen recovery factor was 98.5% with a purity of 99.5%. During the experiment the permeability of the membrane was reduced due to coke formation on the surface. In 2015, Brunetti et al. [85] tested for 2100 h a PdAg membrane (3.6 μm thick) supported onto an α-Al_2_O_3_ support in a packed bed reactor for WGS. Commercial catalytic pellets based on Fe-Cr were employed at 350–400 °C. The largest CO conversion (96.5%) was achieved at 400 °C, 4 bar transmembrane pressure difference, a H_2_O/CO ratio of 3.8 and a gas hourly space velocity 2500 h^−1^ (GHSV). The recovered hydrogen under this condition was around 83%.

**Figure 2:**
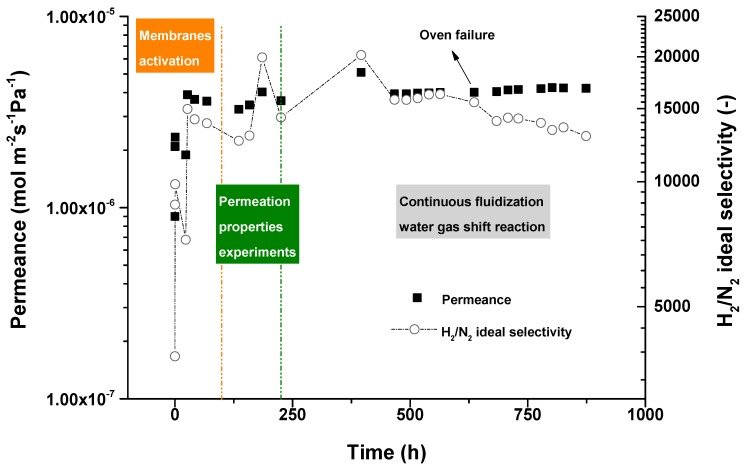
Long-term performance of a fluidized-bed membrane module during 900 h of continuous operation at high-temperature WGS conditions. Reprinted from [86] with permission from MDPI.

A fluidized bed membrane module with a capacity of 1 Nm^3^/h of ultra-pure hydrogen production via high temperature WGS was recently demonstrated by Helmi et al. [86]. The performance of the module was tested under different gas mixtures and WGS conditions during 900 h, showing a very stable performance. As shown in Figure 2, the membrane was first activated and the permeation properties were tested. Then a Pt-Al_2_O_3_ catalyst was integrated in the reactor and a WGS mixture was fed (10 vol. % CO, 30 vol. % H_2_O and 60 vol. % N_2_). During a long-term test at 400 °C and 1 bar of transmembrane pressure difference, perm-selectivities up to 21,000 were measured with a CO concentration at the permeate side below 10 ppm. The hydrogen permeance through the Pd_85_Ag_15_ membranes (4 μm thick) was 3.89 × 10^−6^ mol∙m^−2^∙s^−1^∙Pa^−1^ under the conditions mentioned above, while 95% of the CO was converted. As can be seen in Figure 2, as a consequence of an unexpected oven failure, the N_2_ leakage started increasing due to a high thermal shock and from that moment until the end of the long-term test, the H_2_/N_2_ perm-selectivity decreased. However, the selectivity was still above 12,000 which means that purity of the recovered hydrogen was high enough to feed directly to a fuel cell.

Faroldi et al. [55] compared a commercial Pd-Ag supported membrane with an in-house composite membrane with a 20 μm thick Pd layer prepared by electroless plating onto a modified PSS with NaO zeolite in a fixed-bed MR under different gas mixtures: dry (DRM) and combined reforming of methane (CRM) using Ru as catalyst supported onto La_2_O_2_CO_3_. The best results were obtained for the commercial membrane where the recovered hydrogen at 450 °C was around 80% with a purity of the produced H_2_ of 99.5%. Methane reforming was also studied in fluidized bed membrane reactors using Pd based membranes by Chen et al. [58], who reported methane conversions under ATR conditions in the range 26%–41% in a pressure range of 2600 kPa and reaction temperature between 500 and 600 °C, with a hydrogen yield (mol H_2_/mol CH_4_) ranging from 0.3 to 0.7. In the work reported by Mahecha-Botero and co-workers [59] 25 μm thick Pd-Ag foils (manufactured by Membrane Reactor Technologies, Ltd., Vancouver, BC V6C 1S4, Canada) were sealed onto porous metallic substrates modified with a thin ceramic barrier and tested in a FBMR using NiO and noble metal catalysts supported on alumina for SMR and ATR experiments. Larger methane conversions were obtained under ATR conditions with an O_2_/CH_4_ ratio of 0.35 at 550 °C and a reactor pressure of 900 kPa, having a maximum conversion of 80.9% while the permeated hydrogen purity was 99.988%. In case of operation under SMR conditions at the same temperature and pressure as the ATR experiments, the obtained methane conversion was 73.1% with a recovered hydrogen purity of 99.94%.

Medrano et al. demonstrated that the absence of interactions between a Pd-Ag membrane and Ni/CaAl_2_O_4_ catalysts after several reactive tests under SMR and ATR conditions in a fluidized bed membrane reactor [62]. The authors reported methane conversions above 70% and 80% under steam and autothermal reforming conditions at 600 °C, 3 bar pressure difference and a S/C ratio of 3. Up to 27% and 34% of hydrogen was recovered for steam and autothermal reforming under the conditions used by the authors. The purity of the recovered hydrogen was over 97%, while the ideal perm-selectivity decreased from 574 (before the reactive tests) to 132 (after the SR and ATR experiments). This decrease was attributed to an increase in nitrogen leakages, since the membrane was tested and found stable during 800 h in the temperature range of 500–600 °C before the reactive tests.

Fernandez et al. [63] did not find chemical interactions between the Ru/Ce_0.75_Zr_0.25_O_2_ catalysts and the Pd-Ag (4 μm) membrane during tests in a fluidized bed MR at 550–600 °C. However, pinholes were observed at the membrane surface after 7 days of testing at 600 °C. During experiments carried out under steam methane reforming conditions (S/C = 3 and 1.3 bar) the authors reported 76.4% and 89.35% of CH_4_ conversion at 500 °C and 600 °C, respectively. The purity of recovered hydrogen (20% at 550 °C and 23% at 600 °C) was ~99.98. Small amounts of CO in the permeate stream (120 ppm and 200 ppm) were observed. On the other hand, a larger CO concentration (500 ppm) was measured under autothermal conditions at 600 °C, while a higher methane conversion and hydrogen recovery was obtained, 96.7% and 35%, respectively.

The stability of a 5 μm thick Pd-Ru membrane (0.3 wt. %) was investigated at 580 °C in a membrane reactor for methane steam reforming for 1000 h by Abu El Hawa and coworkers [60]. The membrane was tested at 2900 kPa with a steam-to-carbon ratio of 3 using a Ni-based catalyst. During the experiment the recovered hydrogen remained almost equal (>85%) with a methane conversion of 80%. The purity of the recovered hydrogen decreased slightly during the test. Nevertheless, it remained above 93%. Concerning the impurities at the permeate side, the level of CO_2_ increased from ~2.3 mol % to ~5 mol %. The methane level also increased after 900 h from ~1 mol % to ~2 mol %. On the other hand, CO levels decreased from ~1.7 mol % to almost 0.

Roses et al. [61] performed steam reforming over a catalytic partial oxidation (CPO) catalyst during 260 h in a FBMR at temperatures around 550–630 °C and reactor pressure of 3.5–4.4 bar. Double layered PdAg membranes (from REB) used for hydrogen extraction were deposited by ELP onto a porous metal substrate reinforced with Inconel. The authors studied the effect of the WHSV, pressure and temperature on the methane conversion. The conversion reached in the different experiments at higher temperatures was between 65% and 85%, except for the experiment performed at 550 °C, where a lower methane conversion was achieved (<50%). A higher temperature results in a larger hydrogen flux through the membrane, while a higher WHSV results in a lower conversion due to the reduced gas residence time and increased bubble-to-emulsion mass transfer limitations. The GC (resolution >10 ppm) analysis did not show any presence of CO in the permeated hydrogen, which could therefore be directly used as feed to a PEM fuel cell.

#### 2.1.2. Methanol as Feedstock

Methanol is an interesting feedstock for hydrogen production, especially as it can be considered as a hydrogen storage medium. Iulianelli et al. [87] recently reported a review on methanol steam reforming performed in different MRs. After this review was published, few more experimental studies were reported. In particular, Liguori et al. [64] reported the use of a supported Pd (~7 µm) membrane layer onto a porous Al_2_O_3_ support prepared at Nanjing University of Technology by electroless plating for methanol reforming in a PBMR at 280–330 °C at low pressures (150–250 kPa). The authors used commercial Cu-Zn catalysts supported on Al_2_O_3_ with a steam-to-methanol molar ratio of 2.5. The methanol conversion at 330 °C increased from 75% to 85% when the operating pressure was increased from 150 kPa to 250 kPa. At these conditions, the measured hydrogen yield ranged between 57% and 82%. The permeated hydrogen had a low content of CO (10 ppm), where the hydrogen recovery at 330 °C was improved from around 25% at 150 kPa up to 40% at 250 kPa. The same catalyst was employed by Ghasemzadeh et al. [22] in a PBMR using commercial tubular membranes produced by ENEA (50 µm thick Pd_77_Ag_23_) for recovering hydrogen produced at 280 °C and 2.5 bar with a steam-to-methanol ratio of 3. A total methanol conversion was achieved with 46% of produced hydrogen recovered without presence of any impurities. Mateos-Pedredo et al. [65] reported high methanol conversion using in-house CuO/ZnO (Ac 375) catalysts in a PBMR and a 7 µm thick Pd membrane (Nanjing University of Technology). At 300 °C 97% of the methanol was converted at a transmembrane pressure of 1.5 bar, steam-to-methanol ratio of 2.5 and a WHSV of 2.73 h^−1^. The authors reported an increase in the hydrogen recovery at 330 °C from 63% to 72% when the space velocity decreased from 2.73 h^−1^ to 1.37 h^−1^. The purity of the recovered hydrogen was around 91% and did not vary with the WHSV.

**Figure 3:**
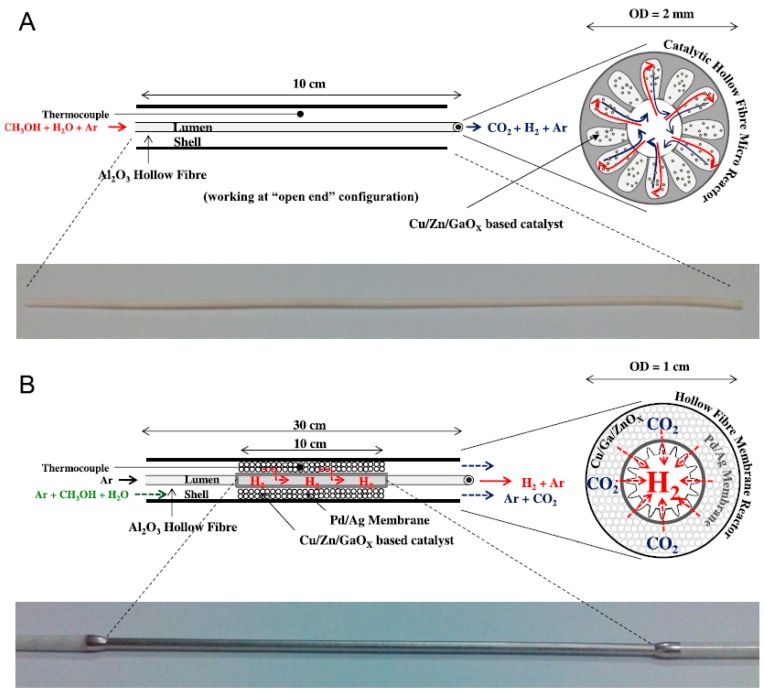
Representation of the catalytic CHFMR (**A**) and HFMR (**B**). Reprinted from [66] with permission from Elsevier.

García-García and coworkers [66] analyzed the performance of two types of Al_2_O_3_ hollow fibres (HF), viz. a catalytic HF micro-reactor (CHFMR) where a Cu/Zn/GaO_x_ catalyst was located in the finger-like regions and a hollow-fibre membrane reactor (HFMR). The hollow fibres for the MR were coated with PdAg by a sequential electroless plating technique growing a 5 µm thick layer followed by annealing in hydrogen at 400 °C for 24 h. In case of the HFMR, a catalyst was deposited on top of the PdAg layer using a co-precipitation method. A schematic representation of the membrane configurations are presented in Figure 3. The methanol conversion of both membrane configurations were compared the conversion in a fluidized bed reactor (FBR) at temperatures between 150 °C and 450 °C and atmospheric pressure. In case of the CHFMR, a low methanol conversion was achieved at 300 °C, around 22.9%, yet much higher than with the FBR, that converted only 4.1% of the methanol fed, producing a four times larger amount of CO in comparison with the CHFMR. Total methanol conversion was obtained at 321 °C and 378 °C in a HFMR and FBR respectively. Those results are related to the presence of the PdAg layer shifting the reaction towards the products as hydrogen was extracted. The permeated hydrogen through the PdAg HF at 400 °C was around 50% of the hydrogen produced during the reaction.

Islam et al. [67] performed their experiments in a fixed bed membrane reactor with a Ni-Zn/Al_2_O_3_ catalyst. The methanol conversion was 78% at 300 °C using a steam-to-methanol ratio of 1, which was four times higher than at 200 °C. Half of the produced hydrogen was recovered through a Pd/PSS membrane prepared by surfactant induced electroless plating (SIEP) at 300 °C. The authors observed carbon deposition on the membrane after being exposed to steam reforming of methanol during 36 h at different temperatures (200–300 °C) in batch mode. In addition, the authors also observed embrittlement of the membrane related to the low temperatures employed.

All these studies suggest that methanol reforming can be successfully carried out in membrane reactors at temperatures below 400 °C. At these conditions complete methanol conversion can be achieved, together with a high hydrogen recovery factor and high hydrogen purity. This makes methanol a very good fuel, or better a hydrogen storage medium, for the hydrogen membrane reactor since the produced ultrapure hydrogen can be directly used in PEM fuel cells.

#### 2.1.3. Ethanol as Feedstock

Steam reforming of ethanol requires higher temperatures than methanol due to larger enthalpy (see Table 1), but requires milder conditions than for methane reforming. The main problem associated with ethanol reforming is the production of undesired by-products, such as formaldehyde, methane, ethylene and carbon, which are formed at moderate temperatures [88]. At operating temperatures around 300–500 °C, complete conversion of ethanol can be achieved, although the methane formation decreases the hydrogen production [89]. For this reason, the ethanol reforming is carried out in membrane reactors at higher temperatures than in conventional reactors in order to reduce the methane formation.

Catalysts investigated for ethanol steam reforming are based on groups 8–11 metals. The activity of many metals supported onto alumina were evaluated by Auprêtre et al. [90] at 700 °C resulting in large conversions in many cases. The best results were obtained with 1% of rhodium, where the hydrogen yield was 72% without the formation of methane. Similar values were obtained with supported nickel catalysts. However, the required amount of this metal is at least 10 times larger. On the other hand, the selection of the support is also important to avoid coke formation and prevent undesired deactivation of catalysts, such as for nickel [91]. Usually, gamma-alumina is used as support, but the high acidity catalyses coke formation. Addition of metals from group 3 (yttrium, lanthanum and scandium) increases the basicity of the supports thereby decreasing coke formation [92], where the highest hydrogen yields were obtained with Rh and RhNi supported onto Y_2_O_3_-Al_2_O_3_ without deactivation after 8 h at 675 °C realizing ethanol conversions of 97.2% and 98.2%, respectively. Rh-based catalysts with a high resistance to carbon deposition and a high activity (giving full conversion of ethanol) were reported by da Silva et al. [68]. Rh/CeO_2_ showed a high stability even under low steam-to-ethanol ratios attributed to the oxygen storage capacity of ceria. The authors also reported results with a Rh/La_2_O_3_-SiO_2_ catalyst, which exhibited also good resistance against carbon deposition [68], related to the capacity of LaO_x_ species to generate lanthanum oxycarbonates (La_2_O_2_CO_3_) [93]. The authors selected Rh/CeO_2_ due to the larger ratio of the permeated hydrogen over the ethanol fed [68]. The recovered hydrogen at 550 °C and a steam-to-ethanol molar ratio of 10 was improved from 15% to 70% when the sweep gas flow rate was increased from 15 mL∙min^−1^ to 130 mL∙min^−1^. 

Nevertheless, cheaper options are required while maintaining a high catalytic activity, such as Co-based catalysts, as suggested by Haga and coworkers [94] who used cobalt supported onto alumina resulting in a high selectivity for ethanol steam reforming.

Dominguez et al. [69] prepared a cobalt talc onto cordierite for ethanol reforming in a PBMR, which was operated at lower temperatures (325–400 °C) favouring the WGS reaction. Total conversion of ethanol was achieved in the temperature range of 350–400 °C and 8 bar pressure using a S/C molar ratio of 3 (steam/ethanol = 6). The hydrogen was recovered through a 30 µm thick PdAg membrane, and the hydrogen recovery was 30% at 350 °C and 7 bar operating pressure and increased to 80% when the pressure was increased to 14 bars.

Iulianelli et al. [70] also reported the use of a Co-Al_2_O_3_ catalyst for ethanol steam reforming in a PBMR, where the hydrogen was extracted via PdAg (50 µm) membranes supported onto a tubular stainless steel module. A 94.2%–94.5% ethanol conversion was obtained at 400 °C in a MR with a retentate pressure of 1.5 bar and a sweep factor of 5.5 (where the sweep factor was defined as the molar ratio between sweep gas (N_2_) and the feed ethanol), whereas in a conventional reactor operated at the same operating conditions a 10% lower of conversion was achieved. The recovered CO-free hydrogen at the permeate side was around 27% for the counter-current mode with a hydrogen yield around 17%–19%. In a second study [71], the ethanol conversion was improved to 100% by using a higher sweep factor of 25.2 (in counter-current mode) and a higher pressure (3 bar). Most of the produced hydrogen was recovered (95%) and the hydrogen yield was increased up to ~60%. 

Espinal et al. [72] showed full conversion of ethanol in a PBMR using cobalt hydrocalcite catalysts doped with potassium supported on cordierite. The authors investigated the influence of operating conditions, such as the temperature, steam-to-ethanol ratio and pressure. The hydrogen was recovered through a 50 μm thick PdAg membrane supported onto a porous stainless steel tube. It was observed that the recovered hydrogen increased from 65% to 82%, when the temperature was increased from 500 °C to 600 °C at the same pressure (12 bar) and S/C ratio (3). 

In bio-ethanol some traces of glycerol and acetic acid could be present, when it is produced by fermentation. Seelam et al. [73] fed directly a mixture of those compounds with water, in a water/ethanol volume ratio of 4, to a PBMR using Co/Al_2_O_3_ and Ni/ZrO_2_ as catalysts. The best results were obtained when using Co/Al_2_O_3_ catalysts at 400 °C and 12 bar, where 94% of the ethanol was converted and 40% of the produced hydrogen was recovered through a Pd (20 µm)/PSS membrane with 5% of impurities in the permeate side. Those results were obtained under a gas-hour-space-velocity (GHSV) of 800 h^−1^. On the other hand, the Ni/ZrO_2_ catalysts showed a higher activity for bio-ethanol conversion at lower pressures (8–10 bar), but the recovered hydrogen was lower than with Co/Al_2_O_3_.

The production of hydrogen from ethanol reforming using Pt-Ni and Pt-Ru as catalysts with a low content of the metals (0.3 wt. %) supported onto nanodiamonds produced by a detonation synthesis technique (DND) in a PBMR was recently reported by Mironova et al. [40]. Defect-free Pd-Ru foils were used as hydrogen membrane. The ethanol conversion achieved at 450 °C and an ethanol-to-steam ratio of 9 was 98%–99%. The obtained hydrogen yield in the MR was 1.5–2 times higher than in a conventional reactor for the same C_2_H_5_OH/H_2_O ratio. The recovered hydrogen through the membranes was around 35%–60% with a presence of 0.01% of impurities. The produced amount of CO with the Pt-Ru catalysts was negligible at 450 °C, but with an increase in temperature some CO was produced. A decline in the permeated hydrogen through the membranes was observed after 50 h on stream (the Pt-Ru catalysts was regenerated every 3–5 h), however, the original hydrogen flux was recovered after a thermal treatment in air at 450 °C.

Full conversion of ethanol was achieved by Murmura et al. [74] with Ni(10%)-Pt(3%) over a CeO_2_-washcoated SiC open cell foam in the temperature range of 340–480 °C. The authors obtained full recovery of hydrogen through a Pd membrane when sweep gas was added over the entire temperature range and pressure difference of 4–8 bar. A small percentage of CO_2_ (0.3%) and CH_4_ (0.2%) was detected in the permeate stream. Iulianelli et al. [75] reached 98% of ethanol conversion at 400 °C, 3 bar and a GHSV of 5000 h^−1^ in a PBMR using Ni (7.5%) CeO_2_ catalysts. The recovered hydrogen (~70%) through a 8 μm thick Pd membrane had a purity of ~80%. Autothermal reforming of ethanol, also referred to as oxidative steam reforming, is another way to produce hydrogen where a small amount of oxygen is added to the feed which allows to operate the reactor in an autothermal regime (see Table 1). Santucci et al. [95] performed the oxidative steam reforming of ethanol in a membrane reactor using Pd-Ag self-supported tubes from ENEA (with a wall thickness of 60 µm) by feeding water/ethanol/oxygen in a molar ratio of 10/1/0.5, where air was used as oxygen source. The largest hydrogen yield (moles of hydrogen permeated per mol of ethanol fed) obtained at 450 °C and a pressure of 200 kPa was 4.1. The authors did not report the ethanol conversion, but an increase in ethanol conversion was observed when the ethanol was diluted with water.

Lim et al. [77] obtained interesting results with PdCu membranes deposited by ELP onto Al_2_O_3_ porous supports from Pall modified with boehmite in comparison with SiO_2_-Al_2_O_3_ membranes obtained by chemical vapor deposition. The authors prepared Na-CO/ZnO catalysts by co-precipitation which was reported to be stable at moderate temperatures during testing periods exceeding 260 h. The best result obtained with 2 µm thick Pd_80_Cu_20_ membranes resulted in an ethanol conversion of 62% at 350 °C and 1 atm. The recovered hydrogen contained some impurities, but the obtained H_2_ purity was around 99.8%. In a study reported later [78], the same authors compared the behaviour of membranes with a Pd (1.3 µm) and PdCu (2 µm) layer deposited onto an α-Al_2_O_3_ hollow fibre by ELP in a PBMR using the same catalyst. Keeping the steam-to-ethanol molar ratio fixed at 13, the authors observed an ethanol conversion of ~74% and ~58% for the Pd and PdCu membranes, respectively, at 360 °C and 1 atm of pressure. Interestingly, the permeated hydrogen flux decreased during the experiments for both membranes, which was attributed to water adsorption on the membrane surface. After 45 h 20% and 60% of the original values (fresh membranes) where measured for the Pd and PdCu membranes, respectively. Additionally, after reactive experiments were carried out, surface damage was observed for both membranes, where larger pinholes appeared on the Pd layer when compared with the PdCu layer. Presence of carbon compounds around the metal was also detected. This study suggested therefore that steam can be interacting with the membrane by adsorption reducing the permeance and concluded that stabilization of the Pd layer (by alloying with other metals) is also required at these moderate temperatures.

Rahman et al. [79] used an YSZ hollow fibre for deposition of a PdAg layer on the shell side by ELP and integrated this into a membrane micro-reactor for ethanol reforming, where a 10 wt. % NiO/MgO-CeO_2_ catalyst was impregnated in the finger like side. The hydrogen yield obtained under atmospheric pressure and 510 °C was 53%. The recovered pure hydrogen in the shell side decreased from 71% to 55% when the temperature was increased from 350 °C to 550 °C. This result indicated that the membrane area per volume of catalyst was limiting at higher temperatures suggesting that lower amount of catalyst could be used in this micro-reactor.

Noble metals such as Pd and Rh over CeO_2_ have shown a high activity for steam reforming of ethanol. López et al. [80] prepared this catalysts with a small amount of these noble metals (0.5 wt. %) for ethanol reforming at temperatures around 550–650 °C. Total conversion of the feedstock was obtained at 650 °C with a steam-to-ethanol ratio of 3 and a reactor pressure of 11 bar. The recovered hydrogen through a PdAg (30 µm)/Inconel membrane under these conditions was 54%. The same catalysts were used by Hedayati et al. [81] in a catalytic membrane reactor. Full conversion of ethanol was obtained at 600 °C. For steam-to-carbon ratio of 1.6, the recovered hydrogen raised from ~17% to ~90% when the pressure was increased from 4 to 12 bar. For the same pressure (12 bar) but higher S/C ratio (3), the recovered hydrogen was ~78%.

All these studies have demonstrated that the application of membrane reactors for ethanol reforming have a very positive influence on the reduction of by-products (especially methane) at lower temperatures. In fact, recovery of the hydrogen through the membrane inhibits the formation of methane and therefore higher yields of hydrogen can be achieved at lower temperatures resulting in higher energy efficiencies.

### 2.2. H_2_ Separation and CO_2_ Capture

While hydrogen can be considered as a clean energy carrier as its conversion to power produces water as the only by-product, the production of hydrogen itself from fossil fuels results in a large amount of greenhouse gases emissions. 

Capture of the emitted greenhouse gases (in particular CO_2_) during hydrogen production and separation is thus an important step to be considered in the life cycle analysis of the hydrogen. Different research groups reported on the possible integration of CO_2_ capture with the hydrogen production using membrane reactors. 

A high degree of process integration is achieved when packed adsorbent-catalyst bed is integrated into a reactor where hydrogen selective membranes are used for hydrogen separation in the so called sorption enhanced membrane reactor (SEMR), as reported by Garcia-Garcia and co-workers [96]. WGS reaction was performed feeding synthetic gas (86.3 mol % Ar, 7.8 mol % CO and 5.8 mol % steam) in the presence of a 10% CuO/CeO_2_ catalyst, obtaining a CO conversion close to 90% at 475 °C. However, the amount of carbon formed at this temperature was quite large (~17%). The recovered hydrogen through a Pd-Ag (ELP) hollow fibre membrane was close to 60%, while simultaneous capture of CO_2_ (44%) was attained using a hydrotalcite-derived Mg-Al mixed oxides. 

Soria et al. [97] reported a hybrid sorption-enhanced membrane reactor (HSEMR) for low temperature WGS. A catalyst (Cu/ZnO-Al_2_O_3_) and CO_2_ sorbent (K_2_CO_3_-promoted hydrocalcite) were packed inside a self-supported 25 μm thick Pd_75_Ag_25_ membrane. Complete CO conversion was achieved at 300 °C and 5.5 bar. Under these conditions, 37% of the produced H_2_ was recovered (with a purity of 100%), while the produced CO_2_ was sorbed by the hydrotalcite. 

When one compares the results of the hybrid sorption enhanced membrane reactor with the corresponding single concepts, i.e., the membrane reactor concept and the sorption-enhanced concept, the main benefits are a double shift effect due to the removal of both CO_2_ and H_2_ at the same time. This results in: (i) higher conversion degrees compared with the single concepts; (ii) higher hydrogen particle pressure due to the higher conversion and removal of CO_2_, which results in higher hydrogen fluxes through the membranes; (iii) higher CO_2_ partial pressure due to the removal of hydrogen and therefore higher CO_2_ capture rates. However, one should also consider the main disadvantages, being: (i) the membrane should experience higher temperatures during the desorption step; (ii) both hydrogen and CO_2_ are now produced at lower pressures and therefore two compression units are required, while in the single concepts only one stream is produced at lower pressure.

**Figure 4:**
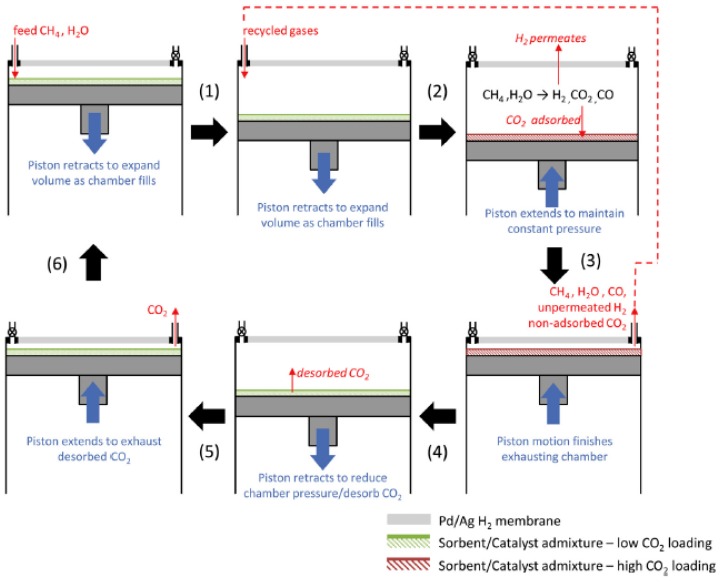
Schematic representation of the CHAMP-SORB reactor for methane SR. Reprinted from [28] with permission from Elsevier.

Another interesting reactor configuration, the CO_2_/H_2_ Active Membrane Piston reactor with CO_2_ adsorption (CHAMP-SORB), with variable volume batch reactor was proposed and theoretically studied by Anderson et al. [28] for methane steam reforming, where hydrogen is recovered via Pd-Ag membranes and CO_2_ is adsorbed. A schematic illustration of the four step cycle reactor is shown in Figure 4. Initially, methane and steam are fed into the chamber where the reaction takes place followed by H_2_ permeation through the membrane and CO_2_ adsorption on the selected sorbent material. To keep the pressure constant during hydrogen permeation and CO_2_ adsorption, a piston moves upwards. The chamber is emptied when the methane reforming is complete with the possibility to recycle the remaining gases for heat recovery. This step is followed by CO_2_ desorption as the piston is moved down resulting on expansion while the temperature is increased to favour the desorption process. Finally, the desorbed CO_2_ is removed from the chamber, as the piston is up-stroking. 

The authors claim that this configuration presents several advantages in comparison with more conventional SEMR, since the sorbent regeneration is carried out in the same unit. However, for steady state gas production several units are required in parallel as in a traditional PSA unit. In a study reported later [98], the same authors demonstrated that the SMR process can be executed at 400 °C with a steam-to-carbon ratio of 2 using a Ni-based catalysts. For hydrogen separation and CO_2_ adsorption a 50 μm thick PdAg layer and K_2_CO_3_ sorbent were used, respectively. The same research group proposed and demonstrated a novel method for portable catalytic reforming of liquid fuels by atomizing the liquid [99]. The so-called CHAMP-Direct Droplet Impingement Reactor (DDIR) was used to generate hydrogen from steam reforming of methanol using a Pd-Ag (25 μm thick foil) membrane and a Cu/ZnO/Al_2_O_3_ catalyst.

For applications where CO_2_ storage is desired, steam is the most convenient purge gas due to its simple condensation from the gas mixture, as was proposed by Reijers et al. [100] using a hydrotalcite as CO_2_ sorbent. Nonetheless, the required amount of steam for total desorption is quite large as the required H_2_O/CO_2_ ratio exceeds 60:1. A system analysis should be carried out to find the optimal operating conditions maximizing the energy efficiency of the system. 

Wu et al. [101] reported a dual-enhanced SMR reactor, were hydrogen was removed with a 7.2 μm thick membrane, while CO_2_ was captured using a nano-CaO-NiO/Al_2_O_3_ sorbent. The CO_2_ sorption capacity of this complex catalyst was 1.6 mol CO_2_/kg at 600 °C and a CO_2_ partial pressure of 20 kPa. The highest methane conversion (98.1%) was obtained at 600 °C with a steam-to-methane ratio of 4. However, since the ideal separation factor (H_2_/N_2_) of the membrane was only 9, the purity of recovered hydrogen was 98.1% with a CO concentration below 1000 ppm. 

**Figure 5:**
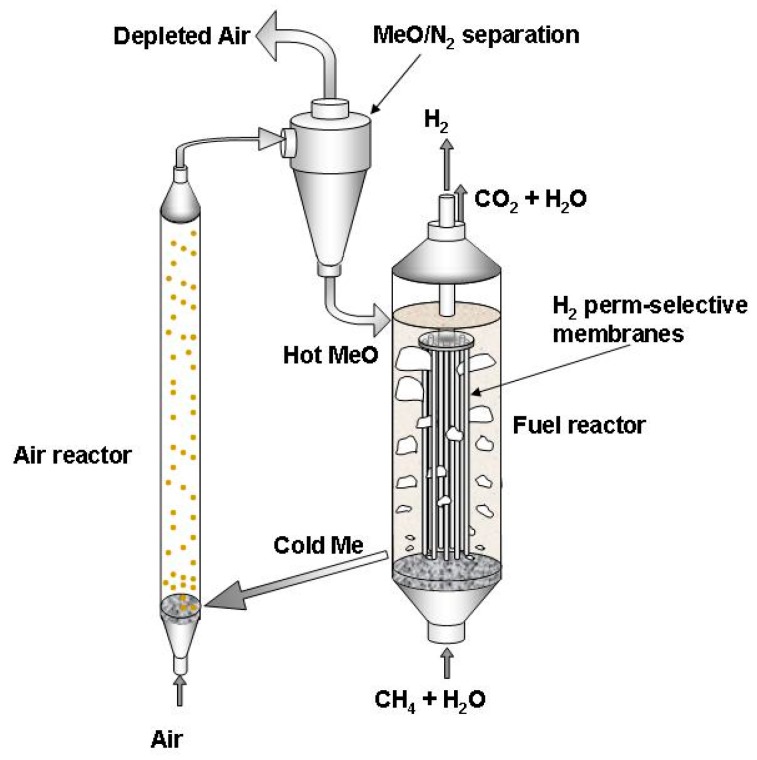
Schematic representation of the MA-CLR system. Reprinted from [102] with permission from Elsevier.

Another novel hybrid concept for hydrogen production and CO_2_ capture has been proposed by Medrano et al. [102], referred to as membrane-assisted chemical looping reforming (MA-CLR). This concept is based on the combination of a fluidized bed membrane reactor with chemical looping reforming (CLR), as schematically represented in Figure 5. The energy required for methane steam reforming is supplied via a chemical looping system, where an oxygen carrier (solid supported metal particle) is oxidized in an air reactor via an exothermic reaction with air. The hot solid is fed to the fuel reactor, where it is reduced to metal and releases the heat for the steam reforming. In the fuel reactor membranes are immersed in the fluidized bed resulting in pure hydrogen production, while the exhaust of the reactor only contains CO_2_ and steam.

When compared with the two single systems, i.e., membrane reactor and chemical looping reforming, the new concept presents several advantages: (i) very high efficiency is obtained at low temperatures; (ii) complete conversion is achieved at temperatures below 600 °C; (iii) much less membrane area is required compared with the single membrane reactor; (iv) a very high CO_2_ capture ratio is achieved because all unrecovered H_2_ and unconverted CO are finally converted at the top of the reactor with a gas-solid reaction with the incoming oxygen carrier [102].

Mahecha-Botero et al. [45] reported CO_2_ capture in a pilot-scale fluidized-bed membrane- reactor using CaO as sorbent mixed with supported Ni on alumina as catalysts for reforming of methane. The pilot plant configuration used 25 µm thick planar PdAg membranes and catalysts as also reported previously by the same authors [59]. Experiments were carried out at 550 °C with a reactor pressure of 300 kPa and a steam-to-carbon molar ratio of 3. The CO_2_ produced during the reforming was effectively adsorbed by CaO producing CaCO_3_. Regeneration of the sorbent was performed by calcination of CaCO_3_ in the regenerator unit separated from the reformer vessel operated at temperatures up to 950 °C. The largest carbon capture observed during these experiments was around 87%. However, the efficiency of CaO to adsorb CO_2_ decreased with time (as expected from the literature on this kind of adsorbent), since the observed levels of CO_2_ inside the reactor started to increase. During the experiments, the purity of the recovered hydrogen was analysed by GC, giving 99.99% purity. The authors also reported the effect of the installed membrane area on the hydrogen recovery and methane conversion producing up to 7.47 × 10^−4^ mol∙s^−1^ of hydrogen. The authors also reported that abrasion of the membrane surfaces was prevented as the calculated superficial gas velocity was smaller than in conventional reformers.

## 3. Characteristics and Properties of Membranes for Hydrogen Separation

As already reported above, the targets for H_2_ membranes are generally taken from the targets set by the United States Department of Energy (US DOE) and some targets set for 2015 have been summarized in Table 3. Some of these targets are within reach thanks to the yearly increasing number of research groups devoted to the further development and improvement of hydrogen selective membranes [6]. The target set for the hydrogen flux for 2015 was 1.135 mol∙m^−2^∙s^−1^ seems quite achievable especially considering the target of 99.99% purity.

However, all these criteria are set for membranes working in the presence of impurities like sulfur compounds and CO, which can be challenging for Pd based membranes (especially the sulfur). The flux target is for a hydrogen differential partial pressure of 20 psi (=138 kPa) with a minimum value for the pressure at the permeate side of 15 psi (=103 kPa), and at 400 °C. The cost per square meter of membrane area seems to be achievable for supported membranes. To surpass the high flux DOE 2015 target, very thin dense membranes are required. The required thickness of the membrane can be estimated from Sieverts’ law for the membrane flux [7,103,104]:

(1) $$J_{H_{2}}=\;\frac{Pe_{H_{2}}}{t}\left(p_{H_{2,ret}}^{0.5}-p_{H_{2,perm}}^{0.5}\right)$$

where $J_{H_{2}}$ is the permeated hydrogen flux, $Pe_{H_{2}}$the hydrogen permeability, t membrane thickness, $p_{H_{2,ret}}^{0.5}$ and $p_{H_{2,perm}}^{0.5}$ are the partial pressures of hydrogen in the retentate and permeate sides respectively [7,103]. To surpass the DOE target, the permeance, defined as the hydrogen flux divided by the driving force, and thus equal to $Pe_{H_{2}}$/*t*, should exceed 3 × 10^−3^ mol∙m^−2^∙s^−1^∙Pa^−0.5^, or 1 × 10^−5^ mol∙m^−2^∙s^−1^∙Pa^−1^ in units that are often used in literature for ease of comparison. With a relatively high permeability of 1 × 10^−12^ mol∙m^−1^∙s^−1^∙Pa^−1^ the required membrane thickness to reach the DOE target is in the order of 0.1 µm, clearly indicating the necessity to develop ultra-thin Pd membranes or layers on supported membranes.

### 3.1. Supported Membranes for High H_2_ Flux

#### 3.1.1. Selection of Porous Support

For the preparation of very thin and pinhole-free supported membranes the choice of the porous substrate is crucial. Different support configurations and materials are commercially available, however, asymmetric tubular supports are preferred because of their larger surface/volume ratio compared with disk shape substrates. The characteristics of the support determine the required selective layer thickness in order to obtain a defect-free thin layer. Both ceramic and metallic supports have been extensively used. On the one hand, ceramic supports are very interesting because of their suitable surface properties and chemical compatibility with Pd layers. However, their integration in a membrane reactor is more complex and they are mechanically weaker than metallic supports. On the other hand, the thermal expansion of ceramic supports is smaller than metallic supports and is closer to the thermal expansion of Pd and its alloys. The thermal expansion coefficients of some materials used for the preparation of composite membranes have been listed in Table 4. The most widely used support is alumina, which has smaller thermal expansion coefficient than Pd, which could cause failures in the membranes when used at higher temperatures. This problem can be reduced by using ZrO_2_ or YSZ (yttria stabilized zirconia), which both have a thermal expansion coefficient closer to that of Pd. 

The most important characteristics of porous supports for the deposition of very thin pinhole-free selective layers are: (a) small pore size; (b) smooth surface (c) thermal and chemical stability.

Supports with a small pore size are required for thin membranes without defects. Thus, the pore size of the support determines the minimum amount of material required to completely cover them and form a continuous surface. Mardilovich et al. [108] have proven that the thickness required to obtain a dense layer is three times the diameter of the largest pore in the support. The minimum thickness of palladium required to deposit a dense layer on top of a porous support with different pore sizes was found to be much larger, as reported by Uemiya [109] (see Table 5). 

Another property of the porous supports influences the required layer thickness: the surface roughness. High surface quality (small roughness) and controlled porosity can be obtained in ceramic supports. High surface quality (small roughness and pore size) can also be obtained by depositing a meso-porous layer onto porous supports [110,111,112], which could however reduce the hydrogen permeation because of the induced mass transfer resistance of the additional layer. Additionally, the adhesion between the Pd layer and the additional meso-porous layer could be a problem, which would result in weak Pd membrane. McCool and co-workers [110] improved the surface quality of commercial α-Al_2_O_3_ by deposition of a 5 µm thick γ-Al_2_O_3_ layer using a slip-casting technique of boehmite. The initial average pore size of the porous support was reduced from 200 nm to 4 nm, with a final porosity around 40%. Very thin PdAg layers (163–525 nm) with different silver content were deposited by DC sputtering. The best permeation results were obtained for a 117 nm thick Pd_82.6_Ag_17.6_ layer, for which the measured H_2_/He selectivity and H_2_ permeance at 300 °C were ~3800 and 7.69 × 10^−8^ mol∙m^−2^∙s^−1^∙Pa^−1^, respectively [110]. Thoen et al. [112] used commercial asymmetric α-Al_2_O_3_ tubes coated with ZrO_2_ from Pall Corporation with a pore size of 20 nm. A 1.3 µm thick Pd_95_Cu_5_ layer was deposited by sequential electroless plating without the presence of EDTA as complexing agent. The permeated hydrogen flux at 365 °C with a feed pressure of ~435 kPa (~63 psig) was 1.5 mol∙m^−2^∙s^−1^. Checchetto et al. [113] deposited a SiO_2_ nanolayer (100 nm) on top of an Al_2_O_3_ porous disk with nominal pore size of 200 nm followed by deposition of 150 nm of PdAg by pulsed laser deposition, which was not completely defect free since the selectivity observed at 300 °C was around 600–900 at a pressure difference of 1 bar with a permeance of 1.4 × 10^−6^ mol∙m^−2^∙s^−1^∙Pa^−1^. Pan et al. [114] modified α-Al_2_O_3_ hollow fibres with an initial pore size of 200 nm with 1 µm thick γ-Al_2_O_3_ by dip-coating and finally 2–3 µm of Pd were coated by ELP with a final selectivity exceeding 1000 at 400 °C and a permeance of 1.1 × 10^−6^ mol∙m^−2^∙s^−1^∙Pa^−1^, which is one order of magnitude smaller than the original support (2.1–2.5 × 10^−5^ mol∙m^−2^∙s^−1^∙Pa^−1^). Wu et al. [115] and Li et al. [116] modified α-Al_2_O_3_ (0.1 µm pore size) and Al_2_O_3_-ZrO_2_ (0.2 µm pore size) supports, respectively, with TiO_2_ followed by photo-catalytic deposition of Pd thin layers of 100 nm and 400 nm, respectively. The observed permeance and selectivity are presented in Table 6, which can be compared with other layers deposited onto modified Al_2_O_3_, and ZrO_2_ supports.

Pacheco Tanaka et al. [111] modified tubular α-Al_2_O_3_ substrates with an average pore size of 150 nm by deposition of a meso-porous γ-Al_2_O_3_ layer by vacuum assisted dip-coating followed by a slow drying process at 40 °C and final calcination at 550 °C. These porous supports were also modified with YSZ/γ-Al_2_O_3_ mixtures, which were calcined at different temperatures (500–1000 °C) [111]. The nitrogen permeance of the supports calcined at 1000 °C was close to twice the permeance of those calcined at 600 °C. During the calcination the pore radius decreased, causing larger pore size distributions at higher temperatures (2–9 nm) and a smaller distribution and size at 600 °C (1–4 nm). Both modified supports [111,117] were used for the preparation of pore-filled type membranes (which will be discussed in more detail in the Section 3.5.2.

In addition to the pore size (distribution) and surface roughness, the stability of the support material under reductive environments at high temperatures and possible interactions with the material of the selective layer (in particular Pd) are two very important aspects to be considered in the preparation of supported membranes. Thermal stability of alumina supports in the presence of hydrogen at different temperatures was evaluated by Okazaki et al. [118]. The authors found that alumina interacts with hydrogen at temperatures above 650 °C producing aluminium. This diffuses through the Pd layer forming an alloy at the interface as revealed by EDX analysis. In their experiments the hydrogen flux was diminished by a factor two after 50 h. This phenomenon proceeds even faster at higher temperatures, as the hydrogen permeation was totally suppressed after 2–3 h at 850 °C. 

Paglieri et al. [119] observed a similar decrease in the permeated hydrogen flux at 750 °C, where Pd was deposited onto alumina porous supports, with a complete and irreversible flux decay that could not be recovered even after thermal treatment in air or hydrogen in order to restore the hydrogen permeation.

**Figure 6:**
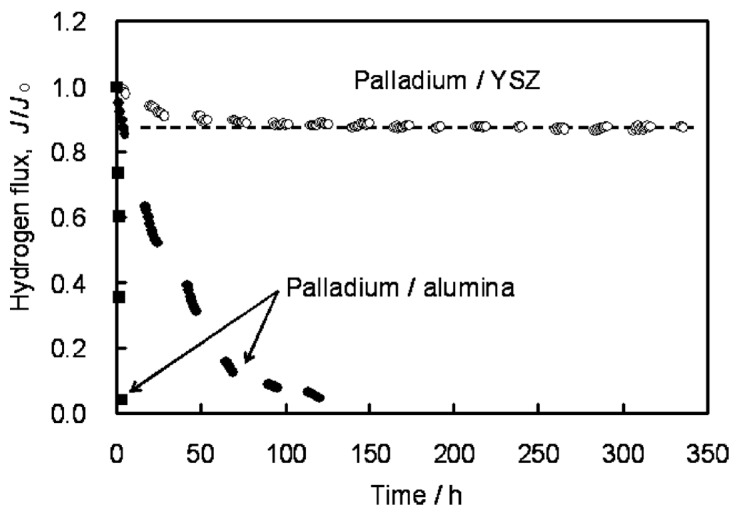
Hydrogen flux measured at 650 °C for Pd-YSZ and PdAl_2_O_3_ membranes at 100 kPa of pressure difference. Reprinted from [121] with permission from Royal Society of Chemistry.

Huang et al. [120] detected diffusion between Pd and TiO_2_ used as interdiffusion barrier. In membranes used for 23 days at 600 °C in the presence of pure hydrogen the authors found diffusion of components of the support (and interdiffusion barrier layer) at a depth of 2–3 µm along the interface. Diffusion was not observed when using ZrO_2_ and YSZ interdiffusion barriers. Stability under reductive and oxidative atmospheres is obtained with cubic phase YSZ. Okazaki et al. [121] found that YSZ represents the best choice for high temperature environments, such as for membrane assisted reactors for autothermal reforming. The authors tested a Pd/YSZ membrane during 336 h at 650 °C in pure hydrogen observing 10% reduction in the hydrogen flux during the first 50 h, as shown in Figure 6. After this period, the hydrogen flux reached a *plateau* without presence of nitrogen leaks. The tested membrane was analysed by cross-section SEM images and it was found that the selective layer did not show presence of Y and/or Zr. However, when using Pd/Al_2_O_3_ composite membranes, the hydrogen permeation flux was completely lost after ~125 h.

As far as metal supports are concerned, most of the papers published in the last few years have used porous supports made of stainless steel, followed by nickel substrates for higher temperatures. Metallic supports employed in the most recent studies have been summarized in Table 7 including some characteristic parameters of the supports and techniques used for the deposition of the ceramic barriers. Stainless steel porous supports with different pore sizes from Mott Metallurgical Corporation are commonly used, where smaller pore size are preferred due to their smoother surface [54,122,123,124,125]. Commercially available PSS supports are made of stainless steel 316L with different pore size, also called media grade. Typical media grades used are 0.1 µm and 0.2 µm, corresponding to an average pore size of 2–5 µm and 10 µm, respectively (measured by mercury intrusion). Larger media grades, 0.5 µm, are less used due to their larger average pore size (10–20 µm) which requires thicker Pd or interdiffusion barriers to reach complete surface coverage. Porous stainless steel substrates from Pall corporation were used with a pore size of 2 µm [126], while YSZ modified PSS from Pall have also been used for membrane preparation [127,128,129]. Some research groups prepared in-house metallic porous supports. A PSS tubular support was prepared by Straczewski using powder from GKN Sinter Metals [130]. On the other hand, nickel porous support were prepared by sintering commercial nickel powder supplied by Vale Inco Pacific Ltd., Hong Kong (SAR), China [106,131]. For high temperature applications (>550 °C), Hastelloy X (Ni-based) from Mott Corporation is another option [62,132]. Except for the ZrO_2_ modified PSS from Pall, all other metallic supports require an interdiffusion barrier, as discussed in the following section. 

Mechanical failure of supported thin films due to the difference in thermal expansion coefficients between the selective layer and the porous supports can be avoided by deposition of an interdiffusion barrier with a similar expansion coefficient of the dense selective layer. Another possibility was reported by Zhao et al. [133], who prepared a membrane with a modified electroless plating bath mixing a solution containing Pd (II) with a γ-Al_2_O_3_ (boehmite) sol. The porous substrate was coated with this solution in the presence of small amounts of polyvinyl alcohol (PVA) and polyethyleneglycol (PEG). Before calcination at 600 °C, the coated layer was dried during two days at low temperature (5 °C) and 65% relative humidity. Before electroless plating, the membrane was exposed to hydrogen at 500 °C to reduce the palladium. Finally, a 1 μm thick Pd layer was deposited by ELP as the support was activated. The permeated hydrogen flux of the supported membrane was 0.108 mol∙m^−2^∙s^−1^ at 450 °C, but the corresponding ideal H_2_/N_2_ selectivity was very low: 20.

The development of a thin membrane with high permeation and selectivity is therefore not always possible due to the factors discussed before such as support pore size, roughness, etc. In many of the studies presented in this period either a high flux or a high selectivity was achieved. However, Bredesen and co-workers (SINTEF) reported the preparation of Pd-Ag membranes with both very high permeation and selectivity [126]. A 2.8 μm thick Pd_77_Ag_23_ layer was deposited by magnetron sputtering onto a silicon wafer, which was removed and rolled onto a 316L stainless steel tubular porous support. The measured hydrogen flux at 400 °C and 26 bar of feed pressure was 8.64 mol∙m^−2^∙s^−1^ with a H_2_/N_2_ selectivity of 1600. These results were even improved after thermal treatment in air at 400 °C for three days. The hydrogen flux and selectivity measured under the same conditions reported before thermal treatment in air were 18.43 mol∙m^−2^∙s^−1^ and 2900, respectively. The initial hydrogen permeance, 6.48 × 10^−3^ mol∙m^−2^∙s^−1^∙Pa^−0.5^, was enhanced one order of magnitude after thermal treatment in air with a measured permeance of 1.46 × 10^−2^ mol∙m^−2^∙s^−1^∙Pa^−0.5^. A long-term test was performed at temperatures below 375 °C during 85 days in the presence of a 50% H_2_/50% N_2_ feed gas revealing great stability for the first 40 days. However, the nitrogen flux increased from 4.46 × 10^−3^ to 6.32 × 10^−3^ mol∙m^−2^∙s^−1^ at 10 bar feed pressure, while the hydrogen flux was maintained at around 8.78 mol∙m^−2^∙s^−1^. The corresponding H_2_/N_2_ ideal selectivity was 1400. Pinholes of about 0.1–0.3 μm in diameter were observed by SEM on the membrane surface exposed to hydrogen after long-term experiments. The membranes prepared by SINTEF were tested under WGS environment at 400 °C and 26 bar feed pressure [134]. In the presence of a gas mixture containing 57.5% H_2_, 18.7% CO_2_, 18.7% H_2_O, 3.8% CO and 1% CH_4_, the measured hydrogen permeance was 1.1 × 10^−3^ mol∙m^−2^∙s^−1^∙Pa^−0.5^. These membranes exhibited at least one year stability under WGS conditions and mixtures of H_2_-N_2_ at feed pressure of 10 bar.

A short air treatment allows activation (or perhaps cleaning) of the membrane and increases the flux of H_2_. It was suggested by Roa and Way [135] that exposure to air resulted in surface cleaning and enhancement in the surface area for “as prepared” (not totally clean) membranes. This effect depends on the exposition time and temperature. However, this effect is not representative for all Pd-alloyed membranes as Zhang et al. [136] reported in their study where Pd_75_Ag_25_ and Pd_90_Au_10_ membranes were “activated” under air treatment at 300 °C. The measured hydrogen flux before and after air treatment for the PdAu layer were practically the same, while the authors observed an increase in the flux for the PdAg membrane ranging between ~80% at a transmembrane pressure difference of 55 Pa^0.5^, and ~50% at a pressure difference of 180 Pa^0.5^.

#### 3.1.2. Selection of Interdiffusion Layer

The undesired diffusion of components from the porous stainless steel support such as Fe, Cr and Ni to the Pd selective layer at high temperatures is a well-known phenomenon. After diffusion, an alloy with Pd is formed that decreases the hydrogen permeance of the membrane due to the reduction of the solubility and diffusivity of hydrogen into the Pd-alloy lattice; Pd and Fe from the support start forming a solid solution at 500 °C in the presence of hydrogen after 20 h [137]. The amount of iron in the Pd layer interface increases from 2 wt. % to 59 wt. % upon a thermal treatment with a temperature increase from 500 °C to 800 °C. In contrast, silver does not form a solid solution with iron at these temperatures.

In order to avoid the diffusion of elements between the metallic support and the dense selective layer during operation at high temperatures, the deposition of a ceramic barrier is performed on top of the metallic porous support. Moreover, metallic supports having an adequate pore size and roughness for thin Pd membranes are difficult to fabricate. Therefore, the deposition of the ceramic layer also improves the surface quality of the original support allowing the deposition of thinner selective layers. 

The selection of the ceramic material for the interdiffusion barrier layer is a critical point to take into account, since an asymmetric membrane composed of materials with different thermal expansion coefficients could result in a total failure of the membrane during operation especially at high temperatures. Ceramics used as inter-diffusion barrier need to have as similar thermal expansion coefficient as the selective metallic layer and support. The most common barrier material, alumina [123,138,139,140], has a low thermal expansion coefficient compared with ZrO_2_ [108,120,141,142,143,144] or YSZ (yttria stabilized zirconia) [120,125,128,130,145], which both have larger thermal expansion coefficients and closer to Pd and its alloys (see Table 4). Other ceramic layers used as barrier are TiO_2_ [120,130] or CeO_2_ [106,146] which both possess a large thermal expansion coefficient and close to the thermal expansion coefficient of Pd. Oxidation of porous stainless steel supports provide a chromium oxide (Cr_2_O_3_) layer, which can act as an interdiffusion barrier. The thickness of the Cr_2_O_3_ layer is controlled by the oxidation temperature and time [145,147] or by the electrodeposition of Cr followed by oxidation treatment in air [147]. Nickel porous supports are generally modified by the deposition of thin ceramic layers of Al_2_O_3_, ZrO_2_ and CeO_2_. By physical vapour deposition, 0.2 µm thick alumina and zirconia layers were deposited [131], while CeO_2_ (0.5 µm) was obtained by dip-coating after modification of the Ni-support by wetness impregnation with alumina [106]. 

The composite membranes based on metallic porous supports modified with interdiffusion barriers have been summarized in Table 8.

The large pore size of commercial PSS supports can be reduced before deposition of ceramic layers by a pre-treatment based on polishing and then etching with an acid solution (HNO_3_ and HCl) as Li et al. suggested [139]. The initial roughness of PSS supports with media grade of 0.2 μm was reduced from 20 μm to 5 μm by polishing. However, most of the pores were completely closed, decreasing more than 99% the initial H_2_ permeation rate. Acid etching of the polished surface removed more than 15 μm of material, opening pores and, as a result, the permeance of the support was enhanced 15% more than with the initial support without polishing. The remaining roughness was reduced by deposition of a 2 μm thick alumina layer using particles of around 2.5 μm. The surface roughness was decreased from 5 μm to 2 μm after deposition of the first layer, which did not cover the metallic substrate completely and the permeation rate was reduced by 40%. Subsequently, the metallic support was completely covered by deposition of a second layer (~1 µm) with a particle size of 0.3 μm. The measured separation factor H_2_/N_2_ was less than 3.74 (Knudsen diffusion), suggesting still a contribution of viscous flow. A defect-free Pd layer (5 μm) was deposited by ELP [139] exhibiting a permeance of 3.39 × 10^−3^ mol∙m^−2^∙s^−2^∙Pa^−0.5^ at 550 °C and a pressure difference of 340 kPa.

A similar method for the deposition of alumina was reported by Chi et al. [140] without pre-treatment for supports with the same characteristics as used by Li et al. [139]. The particle sizes of the deposited layers were about 10 µm and 1 µm. The authors did not report the thickness of the layers, but suggested that the first layer blocked the large pores of the metallic support, while the second layer covered the surface completely [140]. The measured helium flux was reduced 7.36% and 21.3% after deposition of the two layers, respectively. The deposited dense Pd-layer by ELP onto the original support and the modified support with two layers was reduced from 31.5 µm to 4.4 µm. The permeance of the thinner membrane at 500 °C and a pressure difference of 800 kPa was 2.94 × 10^−3^ mol∙m^−2^∙s^−1^∙Pa^−0.5^, with an ideal selectivity H_2_/He of 1124. Additionally, the hydrogen flux through unmodified PSS with a 31.5 µm thick Pd layer was decreasing during the experiment due to diffusion of metal elements from the support to the Pd layer. 

Broglia et al. [138] reduced the original roughness of PSS with a nominal pore size of 0.l μm with γ-Al_2_O_3_ by dip coating followed by the deposition of a second layer obtained by hydrolysis of metal-organic alumina. They found that the mesoporous alumina (3–4 μm) layer covered the support completely. However, part of this ceramic layer was dissolved by the Pd-activation solution due to the acidity of the solution. A pinhole-free 11 μm thick Pd layer was deposited by ELP with total coverage of the modified support. Unfortunately, the authors did not report permeation experiments.

Tong et al. introduced a cerium hydroxide solution into a Mott PSS tubular support (particle retention size of 0.2 µm) applying vacuum in the inner side of tube [146]. Two layers of 6 µm and 10 µm of Pd were deposited by ELP, resulting in a H_2_/Ar selectivity of 108 for the thicker layer, while the separation factor of the 6 µm layer was lower (only 14). The defects of this last membrane were repaired by chemical vapor deposition sublimating Pd (II) hexafluoroacetylacetonate. After 3 CVD treatments the overall thickness of Pd was around 6.4 µm and the selectivity was improved to 565 with a hydrogen permeation flux of 0.235 mol∙m^−2^∙s^−1^ at 500 °C and a pressure difference of 100 kPa.

Straczewski deposited YSZ and TiO_2_ onto an in-house PSS substrate produced using powder from GKN Sinter Metals [130]. A smoother surface was obtained before deposition of the interdiffusion layer by wet powder spraying of fine particles, followed by deposition of <100 µm thick YSZ layer by atmospheric plasma spraying (APS) or thinner (<15 µm thick) TiO_2_ by wet powder spraying (WPS). The thinner TiO_2_ layers presented a larger surface quality (small pore size, large porosity and smooth surface) than the YSZ layers. Furthermore, the measured nitrogen permeance for the TiO_2_ layers was ~1.8 × 10^−5^ mol∙m^−2^∙s^−1^∙Pa^−1^, one order of magnitude higher than for the thicker YSZ layer, ~3.2 × 10^−6^ mol∙m^−2^∙s^−1^∙Pa^−1^. No significant changes were observed on the permeated N_2_ flux through the YSZ layer after nine thermal-cycling tests at 700 °C with different heating rates. On the other hand, the N_2_ flux was reduced every cycle for TiO_2_ till 10% of the initial N_2_ flux. Thicker Pd layers were required and were deposited onto YSZ (24.8–30 μm) than on TiO_2_ (8.3–14.9 μm) due to the better surface quality of last one. A slight increase in the N_2_ flux was measured for the Pd-YSZ-PSS composite after a thermal treatment at 700 °C. Meanwhile, the N_2_ flux decreased for the Pd-TiO_2_ system when the heating rate was increased, suggesting sintering of the particles of the intermediate layer. The properties of the intermediate and selective layers are reflected in the hydrogen permeation flux. The thicker Pd(25.3 μm)-YSZ-PSS membranes permeated 0.14 mol∙m^−2^∙s^−1^ of H_2_ at 600 °C and a transmembrane pressure of 2 bar, in terms of permeance (5.7 × 10^−4^ mol∙m^−2^∙s^−1^∙Pa^−0.5^) one order of magnitude smaller than for the Pd(14.3 μm)-TiO_2_-PSS composite (2.6 × 10^−3^ mol∙m^−2^∙s^−1^∙Pa^−0.5^). The calculated ideal H_2_/N_2_ selectivity was similar and close to 200 for both membranes [130].

Huang et al. [120] compared three different ceramic barriers (ZrO_2_, YSZ and TiO_2_) deposited onto 310L porous supports by different deposition techniques. Very thin (2 µm) ZrO_2_ layers were obtained by magnetron sputtering resulting in incomplete coverage of the porous support, which resulted in intermetallic diffusion at points where the Pd-layer was in contact with stainless steel. Thicker (10–70 µm) and rougher YSZ layers were obtained by APS, where the largest pore size (1.9 µm) of the original support was reduced to 0.78 µm. However, the nitrogen flux was reduced to 15% of the flux through the original support. In contrast, the nitrogen flux of the deposited TiO_2_ by WPS with a large thickness (40–60 µm) was still 77% of the initial value having a smaller pore diameter (0.22 µm). Nevertheless, adherence of the TiO_2_ layer was compromised at higher temperatures. The required thicknesses of Pd to obtain complete coverage via ELP were 9 µm, 14 µm and 23 µm for TiO_2_, ZrO_2_ and YSZ, respectively. Permeance values of the composite membranes at 500 °C and a pressure difference of 0.5 bar are reported in Table 8, where the best results were obtained for a Pd/TiO_2_/PSS with 1.91 × 10^−3^ mol∙m^−2^∙s^−1^∙Pa^−0.5^ with an ideal H_2_/N_2_ selectivity of ~800. Other composites showed a lower permeance related to the thicker Pd layers, however, the selectivity of both was around ~200 Tardini et al. [123] compared the effect of ZrO_2_ and Al_2_O_3_ as an interdiffusion barrier layer on top of PSS, requiring at least a 10 μm thick selective layer (Pd_92_Au_8_) for ZrO_2_ modified porous stainless steel support, while a double thickness was required to deposit a defect-free membrane on top of an Al_2_O_3_ modified porous support. Moreover, 4%–5% Fe was detected in the PdAu layer deposited on top of an Al_2_O_3_ modified composite due to interdiffusion of elements from the metallic support, because the ceramic layer was too thin and did not cover the metallic support adequately. In terms of permeation rates, the Korea Institute of Energy Research (KIER) reported that ZrO_2_ deposited by sputtering was preferred as interdiffusion barrier layer on top of porous nickel supports (PNS), since the measured nitrogen flux was 1.5 times higher when compared with Al_2_O_3_ interdiffusion barrier layers. Larger pore sizes were observed by SEM on the ZrO_2_ supported surface [131]. Chotirach and co-workers [142] reported that a 0.5 μm thick ZrO_2_ (used as a barrier) deposited by DC magnetron sputtering and oxidized in air, reduced the diffusion of elements from the support to the selective layer. The same authors also deposited ZrN, however larger hydrogen permeation rates were obtained with ZrO_2_.

Wang et al. [143] used a colloidal zirconium oxide (colloid size around 3 media grade at a pH 7.6) solution into PSS with media grade of 0.2 μm. The deposition was repeated five times until a constant separation factor between He/Ar was obtained. A pinhole-free Pd layer of 10 μm was deposited by ELP exhibiting 6.86 × 10^−4^ mol∙m^−2^∙s^−1^∙Pa^−0.5^ as permeance at 550 °C and a pressure difference of 100 kPa. Argon was detected during the heating process indicating the formation of defects. 

Gao and co-workers [144] deposited Pd-doped zirconia sol by dip coating onto PSS substrates (0.2 μm media grade) followed by co-deposition of pinhole-free Pd_84_Cu_15_ layers of 5 μm by electroless plating. The thicknesses of the ZrO2 layers were not reported. Homogeneity of the selective layer was confirmed by XPS analysis after a thermal treatment at 480 °C in H_2_ for 5 h. The measured hydrogen flux at 480 °C and a pressure difference between the retentate and permeated side of 250 kPa was 0.6 mol∙m^−2^∙s^−1^ corresponding to a permeance of 2.19 × 10^−3^ mol∙m^−2^∙s^−1^∙Pa^−0.5^.

To select the powder that provides smaller surface roughness, the research group from Universidad Rey Juan Carlos (URJC) [125] studied and reported the porosity and roughness of the intermediate layer of different commercial YSZ particles obtained by atmospheric plasma spraying (APS) on top of PSS with media grade of 0.1 μm. 

**Figure 7:**
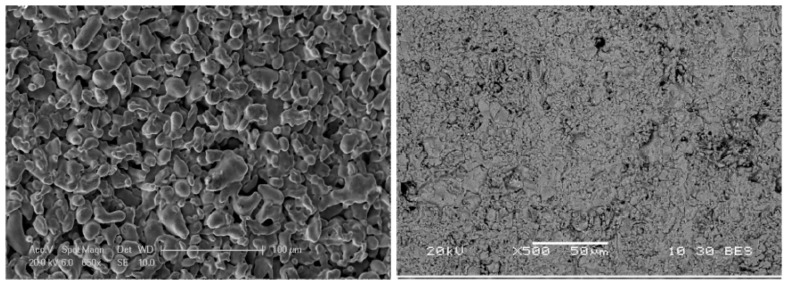
SEM images of original PSS support surface (**left**) and YSZ intermediate layer obtained with Nanox s4007 (**right**). Reprinted from [125] with permission from Elsevier.

The initial porosity of the support (20%) was reduced to 2% by the intermediate YSZ layer made by Nanox s4007 and AMDRY 6600. The selected YSZ powder was Nanox s4007 since the deposited 100 μm thick layer presented the smallest average roughness (Ra = 2.89 μm) compared to the value measured with AMDRY 6600 (Ra = 4.73 μm) and original supports (Ra = 5.87 μm). The differences in surface quality of the original porous support and YSZ-modified with Nanox s4007 are shown in Figure 7. It is clear from the pictures that the layer completely covered the support and at the same time a reduction in pore size was achieved. A 13.6 μm thick Pd-membrane was deposited by ELP onto YSZ/PSS. No nitrogen leaks were detected during single gas tests of the Pd-composite membrane, where the best hydrogen flux measured at 400 °C and a trans-membrane pressure of 2.5 bar was 0.062 mol∙m^−2^∙s^−1^. This flux dramatically decreased in the presence of CO and CO_2_ during gas mixture tests with 70 mol % H_2_. The initial H_2_ flux was, however, recovered after removing all gas mixtures, thus the reduction is to be attributed to the very well-known CO poisoning of the membrane surface [82]. 

Zhang et al. [145] pre-treated PSS (316L) disks with large media grade (0.5 μm) by polishing with #3000 carborundum sandpaper followed by oxidation at 800 °C during 8 h. The measured nitrogen permeance at room temperature was 3.13 × 10^−6^ mol∙m^−2^∙s^−1^∙Pa^−1^, which was two orders of magnitude smaller than for the original support. A larger N_2_ permeance was obtained (1.21 × 10^−5^ mol∙m^−2^∙s^−1^∙Pa^−1^) with thick YSZ layers of 2.5–3 μm deposited directly onto the supports by dip-coating. Diffusion of components from the porous support was suppressed only with YSZ at 700 °C. An 11 μm thick Pd layer was deposited onto an YSZ/PSS substrate by ELP technique. The hydrogen permeance of the composite was 1.05 × 10^−3^ mol∙m^−2^∙s^−1^∙Pa^−0.5^ at 650 °C and a pressure difference of ~220 kPa. The ideal H_2_/N_2_ selectivity was not reported, however N_2_ was observed at room temperature and 100 kPa of pressure difference (10^−1^ mol∙m^−2^∙s^−1^∙Pa^−1^). On the other hand, a thicker Pd layer (25 μm) was deposited onto oxidized PSS, which suffered from intermetallic diffusion at 650 °C, since the hydrogen flux started decreasing after a few h. The authors suggested high atomic vibrations of elements from stainless steel and palladium, since they were exposed to temperatures above their Tamman temperature (316 L, 560 °C; Pd, 640 °C). The hydrogen permeance was measured at 600 °C and the same pressure difference as for the thinner membrane resulting in one order of magnitude smaller permeance (1 × 10^−4^ mol∙m^−2^∙s^−1^∙Pa^−0.5^) at the same temperature.

Interdiffusion barriers of mixed Al_2_O_3_-YSZ were deposited on top of Hastelloy X (0.2 μm media grade) by APS and powder suspension deposition (dip-coating) [132]. The roughnesses for layers deposited by APS were found to be larger than by dip-coating. A 4–5 μm thick PdAg layer was deposited by ELP on top of a modified support with a ceramic interdiffusion layer deposited by dip-coating, and the measured hydrogen permeance was 7.69 × 10^−4^ mol∙m^−2^∙s^−1^∙Pa^−0.5^ at 400 °C and 100 kPa pressure difference. The measured H_2_/N_2_ perm-selectivity was over 200,000. A long-term test with this membrane was performed by Medrano et al. [62] during 800 h in the temperature range of 500–600 °C. The ideal perm-selectivity was maintained over 200,000 during 795 h. Nonetheless, defects started being formed at high operating temperatures (600 °C) and the ideal perm-selectivity dropped to 2650.

Oxidation of metallic supports allows growth of an Fe-Cr oxide (Cr_2_O_3_) layer in order to prevent diffusion of support elements to the selective layer. Ghabiri et al. [122] oxidized a porous stainless steel 316 L disk (nominal particle retention size of 0.2 μm) in air at 800 °C for 12 h before the depositing a Pd_90.2_Ag_3.6_Cu_6.2_ layer by electroless plating. A very thick selective layer, 40 μm, was required to avoid the presence of defects. The thickness and pore size of the grown oxide was not reported. However, it can be expected to be of the order of nanometers with a small reduction of the initial pore size of the supports. 

Deposition of a 4 times thinner Pd layer (10.2 μm) by electroless plating was achieved by Sanz et al. [54] as the retention size of the used PSS was 0.1 μm. Since the initial pore size of supports is small, the required thickness for the creation of a defect-free dense layer is decreased. Fe-Cr oxide was obtained at lower temperatures (650 °C) also for 12 h. Again the thickness of the Fe-Cr oxide was not reported. The membrane was first tested with pure hydrogen before being used with syngas in a packed-bed membrane reactor. The best permeation results were obtained in pure hydrogen at 400 °C and a pressure difference between retentate and permeate sides of 2.5 bar, obtaining a hydrogen flux of 0.054 mol∙m^−2^∙s^−1^ with an infinite ideal perm-selectivity (H_2_/N_2_), which indicates that the membrane was completely dense and defect-free. 

However, it has also been reported that the generated oxide layer by oxidation in air on top of PSS substrates could not completely suppress intermetallic diffusion, as Samingprai and co-workers found [147]. The authors compared oxidized PSS in air at 450 °C for 6 h with Cr_2_O_3_ layers of different thickness obtained by electrodeposition of Cr followed by oxidation at 700 °C for 6 h in air. Intermetallic diffusion was suppressed for a 2 μm thick Cr_2_O_3_ layer on top of substrates with an average pore size of 0.1 μm. The authors deposited Pd onto the modified supports by ELP until a dense film was achieved. A defect-free layer was obtained with a 32 μm thick layer which exhibited a low permeance due to the large thickness, 5.84 × 10^−5^ mol∙m^−2^∙s^−1^∙Pa^−0.5^ at 500 °C and a pressure difference of 100 kPa.

Summarizing, smooth surfaces are required in order to deposit a very thin selective layer without compromising the permeation rate. Also, it is well established that the mechanical stability of thin films is improved as they are supported. Chemical compatibility (avoiding interdiffusion of elements from metallic supports) and closer thermal expansion coefficients between the materials is necessary for long-term stability of the membranes. 

### 3.2. Embrittlement and Sulfur Resistant Membranes

Hydrogen selective membranes are often made of Pd-alloys. The metals used to alloy the palladium help in decreasing the challenges that can be detrimental for the hydrogen flux and hydrogen purity. The first challenge affecting Pd membranes is the hydrogen embrittlement occurring below 298 °C and 2 MPa of pressure. Below these critical conditions, the so called α-β transition occurs. The α-phase appears at low concentrations of hydrogen (solid solution), and the β-phase is formed at high concentrations of hydrogen (metal hydride). Transformation to the β-phase results in crystal expansion with an increased lattice parameter, and may cause membrane failure. Characteristic lattice parameters and Pd/H ratio of both phases and pure palladium are shown in Table 9 [148,149].

This problem can be diminished by alloying Pd with elements such as silver. For instance, it has been proven that the durability of Pd membranes alloyed with 20% of silver is improved and H_2_ embrittlement was not observed after cycling hydrogenation/dehydrogenation even at temperatures as low as 100 °C [150]. Additionally, it has been proven that the hydrogen permeance is improved by 70% when the silver content reaches 23% in comparison with pure palladium [151]. McCool et al. [110] investigated the effect of the silver content on membrane embrittlement. Membranes with a lower content of silver (4 wt. %) delaminated at 200 °C in the presence of hydrogen, whereas membranes with a larger amount of silver (7.8 wt. %–12.3 wt. %) delaminated at lower temperatures (150 °C). The same authors did not observe peel-off of the membranes at 150 °C when using higher contents of silver. 

Furthermore, alloying palladium with other noble and non-noble metals could enhance the permeation rate, reduce the temperature of phase transition and improve the sulfur resistance [152]. The largest permeance improvement has been reported when alloying Pd with yttrium in a weight content from 6.6% to 10%, exhibiting a hydrogen flux 350%–375% larger than for pure palladium [151].

When integrating the membranes in membrane reactors for steam reforming of different feedstocks (CH_4_, CH_3_OH, C_2_H_5_OH), the membrane surface may be exposed surface to gases like H_2_S and CO which react with palladium and/or other metals, poisoning the membrane and decreasing the permeation rate. 

As far as the CO poisoning is concerned, it has been demonstrated that CO adsorbs on the palladium surface occupying the same sites where hydrogen adsorbs before splitting [82]. The poisoning of the membrane occurs at very low concentrations of CO, and when the surface coverage reaches a certain value, the additional CO in the mixture only contributes to the dilution of the hydrogen. The effect of CO is well studied, and it is evident that this poisoning decreases with temperature (as the adsorption is decreased at higher temperatures). Moreover, the membrane can be completely regenerated by removing the CO, and the CO poisoning effect can be easily taken into account in models by modifying Sieverts’ equation with a Langmuir type sorption term [153]. On the other hand, poisoning with H_2_S is a more serious problem for Pd-based membranes. First of all, the poisoning with H_2_S occurs at much lower concentrations (at ppm levels). Secondly, the poisoning occurs via a reaction and a palladium sulfide layer is produced at the membrane surface that in many cases cannot be removed, so that the poisoning is not completely reversible. Alloying Pd with other metals can also help in decreasing the poisoning effects of the contaminant. Pd membranes alloyed with Ag have been shown to be more resistant to hydrogen inhibition by CO [154,155]. Resistance to H_2_S poisoning is improved by alloying palladium with noble metals. The most common alloys which present poisoning resistance are Pd-Cu [156] and Pd-Au [127] alloys. 

Presence of gold reduces embrittlement problems associated with the hydride phase transformation and improves resistance to sulfur poisoning due to the large heat of formation of Au_2_S (+230.5 kJ∙mol^−1^) compared with other elements of the periodic table, which possess a negative value of heat formation [152]. 

Gade et al. [157] prepared PdAu membranes with different gold contents (0–20 wt. %), where Au was sequentially deposited on top of Pd layers by EDTA-free electroless plating, followed by an annealing treatment at 750 °C for 20 h in order to create an homogeneous alloy. However, heterogeneous layers were obtained by this sequential deposition of Pd and Au [157]. Deposition of gold happens indeed on top of palladium and not on gold surfaces. When the desired amount of gold is larger than the maximum deposited by electroless plating, extra metal was electrodeposited using a commercial electroplating bath from Caswell Incorporation. Different layers were deposited with an increasing content of gold. The best hydrogen permeance was measured for a 5.4 µm thick membrane with 4.1% of Au, 2.05 wt. %) 10^−3^ mol∙m^−2^∙s^−1^∙Pa^−0.5^ before thermal treatment in air with an ideal selectivity H_2_/N_2_ of 2500. However, the permeability of the obtained PdAu layers was lower than the ones obtained by other techniques such as physical vapor deposition or cold-work. This is most probably due to the poor interdiffusion of Pd and gold during annealing in a nitrogen atmosphere, especially when the amount of gold is increased [157]. To better understand this, different annealing processes were tested for a Pd-Au foil with 7.8 wt. % gold under a nitrogen stream for 20 h. XRD diffractograms revealed that after thermal treatment at 400 °C the Pd and Au were not perfectly alloyed. However, a completely alloyed layer was obtained when the temperature was increased to 750 °C. Okazaki et al. [158] tested a 24 h of annealing treatment in hydrogen at 750 °C and obtained full homogenization of a Pd_92_Au_8_ alloy deposited sequentially onto an Al_2_O_3_ porous substrate with an average pore size of 150 nm. Meléndez et al. [159] reported a Pd_79_Ag_16_Au_5_ homogeneous membrane after an annealing treatment at 550 °C for at least 8 h. The authors co-deposited a ~4 μm PdAg layer on top of an α-Al_2_O_3_ support followed by annealing at 550 °C for 2 h. Finally, gold was added to this membrane at 98 °C and annealed at 550 °C to create the alloy. Different tests were performed with different annealing times. The XRD patterns of the samples annealed for 3 and 6 h, showed the peaks corresponding to Pd_79_Ag_16_Au_5_ alloys, while the peak (111) related to Au disappeared. A homogeneous ternary alloy was observed after 8 and 24 h of annealing and this was confirmed by ICP analysis. The measured hydrogen permeance at 300 °C and 1 bar of pressure difference was 1.12 × 10^−6^ mol∙m^−2^·s^−1^∙Pa^−0.5^ with a H_2_/N_2_ ideal separation factor of ~8800. 

Hatlevik and co-workers [128] developed a defect-free 2.3 μm thick Pd_95_Au_5_ membrane which possesses a high hydrogen flux (1.01 mol∙m^−2^∙s^−1^) and ultra-high H_2_/N_2_ ideal perm-selectivity (82,000) at 400 °C and 1.38 bar pressure difference. The Pd_95_Au_5_ thin layer was deposited by sequential electroless plating followed by annealing to produce a homogeneous alloy on top of a commercial porous stainless steel support with an interdifussion barrier of 50 μm thick YSZ deposited by Pall Corporation. 

Tardini et al. developed defect-free PdAu membranes by sequential electroless plating on top of Al_2_O_3_ or ZrO_2_ modified porous stainless steel [123]. A 12 μm thick Pd_92_Au_8_ selective layer (annealed at 500 °C for 120 h in hydrogen) showed its highest hydrogen flux of 0.16 mol∙m^−2^∙s^−1^ at 450 °C and a pressure difference of 100 kPa, with a calculated ideal H_2_/N_2_ perm-selectivity above 10,000. The asymmetric membrane was tested for 250 h in hydrogen (120 h at 10 kPa and the last 130 h at 100 kPa) showing a good stability of the membrane at temperatures between 400 and 500 °C. No experiments in the presence of H_2_S were reported by the authors.

A Pd_97_Au_3_ thin membrane deposited by sputtering using radiofrequency (RF) and direct current (DC) for Au and Pd deposition respectively, was reported by KIER (Korea Institute of Energy Research) [131]. A 200 nm interdiffusion barrier (ZrO_2_ or Al_2_O_3_) was sputtered on top of an in-house Ni disk-shaped porous support. The Ni-support modified with ZrO_2_ was selected due to the larger nitrogen permeation flux. A 3 μm Pd/0.05 μm Au layer was deposited followed by a thermal treatment to alloy the two metals. The best results were obtained at 450 °C and 2000 kPa pressure difference, where the measured hydrogen flux and selectivity were 4.07 mol∙m^−2^∙s^−1^ and 2000, respectively. For lower hydrogen pressure differences, a higher selectivity was achieved, up to 5000 at 100 kPa. The XRD pattern after hydrogen measurement revealed PdAu alloy peaks which were not presented on the diffractogram of the fresh membrane. However, SEM-EDX on the membrane cross section showed higher concentrations of gold close to the interdiffusion barrier, which proved that homogeneity of the selective layer is not completely obtained. 

H_2_S poisoning of Pd is also reduced by the introduction of Cu into the Pd lattice. Depending on the amount of copper in the alloys, the FCC structure is modified to BCC, which is less resistant to sulfur compounds [160]. Ryi and coworkers prepared a sulfur resistant 7 μm thick Pd_93_Cu_7_ layer by magnetron sputtering deposited on top of CeO_2_/PNS (porous nickel support) [106]. First, the PNS surface was modified with alumina particles (300 nm) by wet impregnation of an aluminum nitrate solution followed by calcination at 500 °C for 10 h. An even smoother layer made of alumina and ceria was deposited on top of the alumina-modified PNS, employing nanoparticles ceria sol (<25 nm), using a dip-coating approach followed by calcination at 700 °C in the presence of hydrogen for 2 h. Knudsen diffusion was suggested for the modified support since the measured ideal perm-selectivity of H_2_/N_2_ was 3.4, which is close to value when Knudsen diffusion is dominating (3.74). The observed H_2_/He selectivity for a dense (defect-free) layer was above 50,000 and the measured hydrogen flux at 500 °C and a pressure difference of 400 kPa was 0.28 mol∙m^−2^∙s^−1^. 

Gharibi et al. [122] prepared novel Pd_90.2_Ag_3.6_Cu_6.2_ membranes by sequential ELP, which kept the hydrogen flux constant even after exposing them to 5 ppm H_2_S in a hydrogen stream. On the other hand, the authors demonstrated that binary PdAg membranes suffered a 15% reduction in the initial hydrogen flux due to sulfur poisoning. A Pd-Ag-Pd layer sequentially deposited was annealed at 500 °C and followed by the deposition of a PdCu bilayer and finally annealed at 480 °C in order to form the desired ternary alloy. The membrane thickness was 40 μm where only 0.5 μm was Cu. SEM-EDX of the cross-section revealed a lower content in silver in the first 7 μm of the selective layer (where the PdCu bilayer was deposited). Copper was not detected at a depth larger than 7 μm, so there was not a complete alloy formed between the three elements. The highest hydrogen flux was measured at 280 °C and 90 kPa of difference pressure (using pure hydrogen as feed) and was 0.054 mol∙m^−2^∙s^−1^ and the obtained H_2_/N_2_ ideal perm-selectivity was around 700. 

Zhao et al. [161] observed that total hydrogen flux recovery is possible for Pd_81_Cu_19_ at 500 °C after being exposed to 7 and 35 ppm of H_2_S. During inhibition with H_2_S, reduction of defects was found (i.e., largest H_2_/N_2_ perm-selectivity). This effect might be related to sulfur segregation at the grain boundaries [162]. However, during regeneration, nitrogen leaks doubled and the selectivity halved (from 2369 to 1194) compared with the initial values before poisoning with hydrogen sulfide [161]. The same authors reported [163] that addition of silver (5, 10 and 19 wt. %) to the PdCu alloy increased the inhibition of the hydrogen flux through the membrane, when it is exposed to H_2_S. As a conclusion, sulfide formation is enhanced as the concentration of silver is increased in the ternary alloy. Membranes with 10 wt. % of silver showed in a XRD pattern the formation of Pd_4_S and Ag_5_Pd_10_S in the presence of 100 ppm H_2_S at 500 °C. 

In 2015, Nayebossadri et al. [164] reported that the introduction of zirconium traces into PdCu alloys provides structural stability at high temperatures. After exposure to 1000 ppm H_2_S at 450 °C for 14 h, the hydrogen flux of Pd_65.1_Cu_34.9_ and Pd_61_Cu_37.2_Zr_1.8_ were reduced by 80% and 60%, respectively.

**Figure 8:**
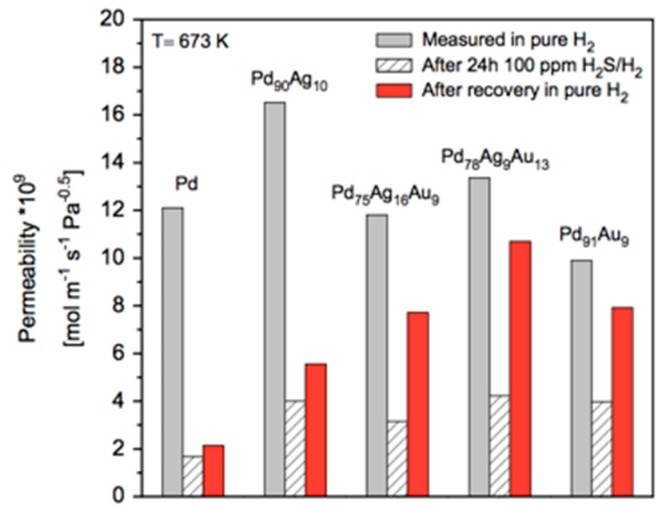
Hydrogen permeability at 400 °C (673 K) measured in pure hydrogen (grey bars), after exposure for 24 h to 100 ppm of H_2_S (cross lined bars) and after removing H_2_S and flux recovery (red bars). Reprinted from [124] with permission from Elsevier.

In a study performed by Braun and co-workers [124], Au was introduced into different PdAg alloys by electroless plating with a similar deposition strategy as was explained for the PdAgCu membranes. An oxidized stainless steel 316L disk was modified with ZrO_2_ deposited by dip-coating assisted by vacuum. After deposition of binary and ternary alloys, a long annealing process (72–120 h) was carried out at 500–600 °C, resulting in a highly homogeneous alloy along the membrane cross-section, as concluded from SEM-EDS after all experiments. The permeability at a differential pressure of 50 kPa for every alloy (~14 μm thickness) were compared at 400 °C in presence of pure hydrogen, after being exposed for 24 h to 100 ppm H_2_S (in hydrogen) and after removing the H_2_S and feeding pure hydrogen (see Figure 8).

As was expected, in presence of pure hydrogen the highest permeability was measured for Pd_90_Ag_10_ alloy followed by Pd_75_Ag_16_Au_9_ > Pd_78_Ag_9_Au_13_ > Pd_91_Au_9_ > Pd. In all cases the hydrogen permeability was reduced when introducing H_2_S, however, the most poisoned materials were pure palladium and Pd_90_Ag_10_ alloys, while a lower decrease was observed for layers alloyed only with gold. The larger hydrogen permeability recovery after H_2_S removal was for the ternary layer with 13% Au, recovering 80% of the permeability before exposure to H_2_S during 24 h [124]. Lewis et al. [127] prepared a Pd_77_Au_23_ (5.7 μm) membrane, deposited by sequential electroless deposition followed by annealing at 500 °C for five days, which showed good permeation results even in the presence of 20 ppm H_2_S in hydrogen, where the hydrogen flux decreased only 29% at 500 °C and the initial flux was totally recovered after removing H_2_S. A larger reduction, 52%, in the hydrogen permeance was observed for Pd_67_Au_20_Ag_13_ (9.3 μm) at the same temperature and H_2_S concentration. When the operating temperature decreased to 400 °C, the reduction in the hydrogen flux was 75%. Both alloys were tested under WGS conditions (49% N_2_, 36% H_2_, 11% CO_2_, 3% H_2_O and 1% CO) in the presence of 20 ppm H_2_S during 96 h resulting in similar behaviour. Before the introduction of H_2_S, the PdAuAg hydrogen permeance at 400 °C and feed pressure of 1.1 MPa was ~1 × 10^−3^ mol∙m^−2^∙s^−1^∙Pa^−0.5^, which is slightly larger than for the PdAu layer (~8 × 10^−4^ mol∙m^−2^∙s^−1^∙Pa^−0.5^). After being exposed for 48 h to syngas, H_2_S (20 ppm) was introduced resulting in a reduction of the hydrogen permeance of both membranes. When removing H_2_S, the permeance started recovering and after 24 h, in the presence of syngas, the initial hydrogen flux was reached.

An interesting study was reported by Lee et al. [165], where a PdAu (1.4 wt. % Au) membrane was covered with 179 nm thick Pt-ZrO_2_ layer. The PdAu layer was deposited by magnetron sputtering on top of a modified porous nickel support. The authors studied the influence of the deposition of a protective layer onto the selective layer. After been exposed to short periods with 100 ppm of H_2_S, the hydrogen flux of the covered membrane decreased to 92% of the initial value at 400 °C, while the uncovered membrane decreased till 80%. The perm-selectivity (1650) of the uncovered membrane declined to zero due to formation of big holes. However, no bulk sulfidation was observed. On the other hand, the selectivity of the covered membrane remained almost unchanged (at 2500) after exposure to H_2_S.

Peters et al. [166] reported the influence of exposure of a PdAg layer (10 μm thick foil on top of a microchannel support) to different compositions of H_2_S in a temperature range of 350–450 °C. The authors observed that inhibition is more influenced by the H_2_S level and temperature than the exposure time. Up to 30% and 80% reduction in H_2_ flux was observed when the membrane was exposed to 2 ppm and 20 ppm H_2_S, respectively. However, the hydrogen flux was almost completely recovered even when the membranes were exposed to 20 ppm H_2_S for 10 min.

Sulfur resistance of PdAu alloys could be improved by alloying with Pt, as Coulter and co-workers [167] reported in their work. Platinum improves the sulfur tolerance at the expense of the H_2_ permeability, which is reduced. The authors showed that the hydrogen permeance of self-supported 33.2 µm thick Pd_77_Au_12_Pt_11_, obtained by magnetron sputtering, was constant during 90 h in the presence of 20 ppm H_2_S. The recovered hydrogen purity was enhanced from 99.7% to 100% when H_2_S was removed. The measured hydrogen flux after exposure to H_2_S at 400 °C and feed pressure of 1.27 MPa was around 0.2 mol∙m^−2^∙s^−1^ in the presence of 50 vol. % H_2_, 30 vol. % CO_2_, 19 vol. % H_2_O and 1 vol. % CO.

### 3.3. Thermal Stability at High Temperature

For Pd-based membranes, one can distinguish “low” (below 450 °C) and “high” (above 450 °C) temperature applications. Many industrially relevant applications fall in the “low” temperature class, including high-temperature water-gas-shift. Other applications, such as methane steam reforming, it would be convenient from an efficiency point of view, to work at higher temperatures (in the range of 600 °C). At these temperatures a good balance between membrane flux, reaction kinetics and thermodynamic constrains can be found. For these “high” temperature applications, the main issue for the membrane is their thermal stability and sealing.

For Pd thin films it was observed that the high-temperature resistance is somewhat limited due to pinholes/defects formation. As temperature increases, not only the hydrogen diffusion through the membrane lattice is improved, but also Pd diffusion itself is increased, which could result in the formation of micro-holes, which are responsible for the loss of selectivity. Strategies to increase the thermal stability are based on employment of: (i) different synthesis techniques; (ii) alloying the metal with different dopants; and (iii) thermal treatments. It was recently observed by Abu El Hawa and coworkers [129] that alloying Pd with Pt or Ru improves the stability of thin films deposited by ELP. This phenomenon is related to the higher melting points of both alloying elements reducing the diffusivity of each atom along the thin film for membranes with a thickness lower than 5 μm. Experiments performed at 500–600 °C showed that the N_2_ leaks increased faster for Pd than for alloys with 27 wt. % Pt or 0.3 wt. % Ru. An increase in the nitrogen leakage was measured at 600 °C for every membrane, but the increase in the N_2_ leakage was one order of magnitude larger for the pure Pd membrane. In terms of hydrogen flux and selectivity, the PdPt alloy represented the best choice, since the hydrogen flux was extremely stable after 850 h, while pinhole formation was very low.

In a recent study of the same authors [60], the stability of a 5 μm thick Pd-Ru membrane (0.3 wt. %) was investigated at 580 °C in a membrane reactor for MSR for 1000 h (described in Section 2.1). After the experiments, the membrane was cooled down to room temperature to check for leakages at the membrane by submerging the membrane in water and pressurizing the membrane from the lumen side with nitrogen to 1 barg. Small bubbles were observed emerging from the membrane surface suggesting the formation of defects during the long term test. The addition of Ru decreased the Pd grain growth, so that the grain size did not reach the selective layer thickness. Addition of ruthenium in small quantities (<1 wt. %) did not only improve the mechanical properties of the alloy, but also the permeability increased [168]. However, due to their immiscibility, alloys of Pd and Ru with higher amounts than 1 wt. % cannot be obtained at temperatures below 600 °C [169]. It was recently reported by Didenko and coworkers [170] that the properties of a Pd-In-Ru alloy (93.5; 6; 0.5 wt. %) showed an almost three times larger hydrogen flux than Pd_77_Ag_23_.

Applications of membranes for high-temperature processes such as methane steam reforming, which are often also operated at elevated pressures, need supports with higher mechanical stability. While metallic membranes are mechanically more stable than ceramic supported membranes, high-temperature applications require metals that are more stable than stainless steel. This is why often Inconel and Hastelloy supports are being investigated. Porous nickel supports (PNS) represents another possibility, however, all metallic supports still require surface modification to avoid interdiffusion [106]. 

### 3.4. Non-Palladium Membranes

**Figure 9:**
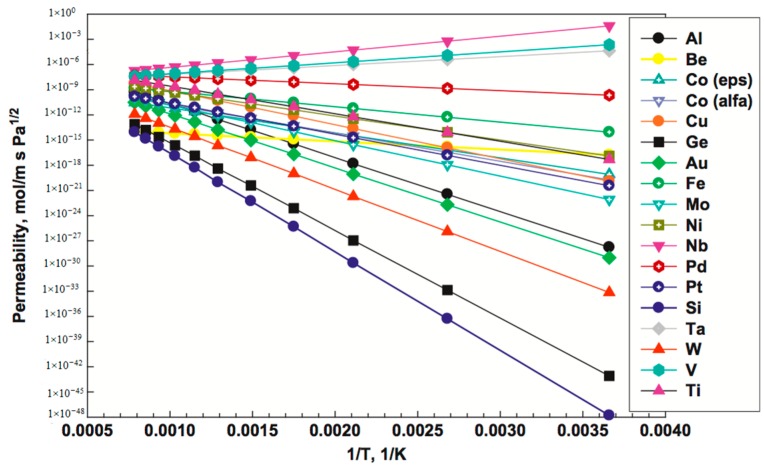
Hydrogen permeability as a function of temperature for different interesting metals. Reprinted from [172] with permission from Elsevier.

Palladium and its alloys are not the only materials proposed for the preparation of hydrogen perm-selective membranes. BCC metals with a high hydrogen diffusion rate and lower thermal expansion (closer to alumina, 7.6 × 10^−6^ °C^−1^) for hydrogen separation and purification have been studied and fully characterized already more than 20 years ago. Elements from group V such as Nb, V and Ta possess higher permeation rates than FCC metals and alloys [171] and their permeation rate increases as the temperature decreases, as shown in Figure 9 [172].

This phenomenon is based on the number and size of octahedral and tetrahedral interstices. The number of tetrahedral interstices per atom in FCC and BCC structures is 2 and 6 respectively, which have a relative size to metallic atom radius of 0.225 and 0.291, respectively. In case of octahedral sites, the FCC structure gives three times more sites than the BCC structure with larger size, but the tetrahedral sites represent the main path for transport of hydrogen through the crystal structure. Despite the higher permeation rates, the main disadvantage of these metals is the low resistance to embrittlement, which leads to membrane failure after only few h of operation [173].

Moreover, their surface activation is much larger than for palladium and the surface is easily oxidized in the presence of low oxygen content, even at very low temperatures (~100 °C) [8]. Their susceptibility to oxidation and their low catalytic activity for hydrogen dissociation/recombination can be solved by covering their surface with other materials which acts as oxygen barrier and as catalysts for hydrogen. Often the membranes based on these metals were prepared by depositing a very thin layer of palladium on both sides of the membrane [174]. However, the embrittlement problem is not solved and remains a key issue for these membranes. Resistance against embrittlement is enhanced by alloying with metals which can reduce the hydrogen solubility. As suggested by Kim et al. [175], addition of Fe to vanadium decreases the hydrogen solubility and the ductility of the alloy. Additionally, the introduction of aluminium to V-Fe alloys increases the embrittlement resistance, but the hydrogen solubility is dramatically decreased. A V-Fe-Al ternary alloy was prepared by arc melting and 400 μm thick disc cut by electrical discharge. In order to promote hydrogen dissociation, palladium was sputtered in both sides (150 nm). The best result was obtained with a ternary alloy V_0.9_Fe_0.05_Al_0.05_ at 500 °C with a permeance of 3 × 10^−4^ mol∙m^−2^∙s^−1^∙Pa^−0.5^, while the embrittlement resistance was enhanced since (under cooling tests) the membrane only failed when operated close to 175 °C. Better results were obtained by Alimov and co-workers [174] with a Pd(2 μm)-V(100 μm)-Pd(2 μm) composite membrane obtained by depositing palladium by electroless plating onto a vanadium disc. At 400 °C the hydrogen permeance was 1.8 × 10^−3^ mol∙m^−2^·s^−1∙^Pa^−0.5^. In order to minimize the cost of the membrane, Viano et al. [176] replaced the Pd layer at the permeate side with nickel. The flux of the asymmetric membrane composed by Pd(500 nm)-V(200 μm)-Ni(150 nm) was 65% of that of a Pd-V-Pd symmetric membrane. On the other hand, Oh et al. [177] showed that addition of yttrium (0.2 at. %) to a V_85_Ni_15_ 500 μm thick layer enhanced the stability of the membrane at 480 °C. The permeability of the V_85_Ni_15_ layer decreased from 2.4 × 10^−7^ to 1.4 × 10^−7^ mol∙m^−1∙^s^−1∙^Pa^−0.5^ after 290 h, while the doped layer with yttrium showed a smaller decline, from 2.0 × 10^−7^ to 1.65 × 10^−7^ mol∙m^−1∙^s^−1∙^Pa^−0.5^.

Watanabe and co-workers improved the embrittlement resistance as well as the hydrogen permeation flux of niobium by alloying it with 5 mol % of W or Ru [178]. Paglieri et al. prepared a ternary (Ni-Nb-Zr) and quaternary (Ni-Nb-Zr-Ta) alloy membranes by planar flow casting, which were coated with 500 nm of palladium by PVD [179]. The thickness of the disk-shaped membranes was around 50–90 μm. Those membranes were tested employing porous stainless steel with 0.5 μm grade as mechanical support. The authors found that increasing the amount of zirconium enhanced the hydrogen permeation flux, while the embrittlement resistance was reduced as well as the thermal stability. When tantalum is added, the thermal stability was improved at the expense of a decrease in permeability. The higher permeability (1.4 × 10^−8^ mol∙m^−1^∙s^−1^∙Pa^−0.5^) was obtained for (Ni_0.6_Nb_0.4_)_0.7_Zr_0.3_ alloy at 450 °C.

### 3.5. Atrittion Resistance Membrane

The durability of the membranes in membrane reactors could be compromised by their contact with catalyst particles. This could even be more stringent when the membranes are integrated in a fluidized bed membrane reactor, as the surface of the selective layer is directly exposed to particles in continuous movement. While very long-term tests should be carried out to assure the stability of the membranes under such conditions, a possible solution to circumvent or decrease erosion problems is to reduce the exposed surface of the membrane. An option for minimization of the membrane area exposed to the fluidized suspension is the use of Cermet membranes, since the exposed area is about 50% of conventional thin-film membranes. Another possibility to protect the surface from contact with catalyst particles is by covering it with a nanoporous ceramic layer, such as in the pore-filled type membranes prepared by Pacheco Tanaka et al. [111]. A comparison of the permeation characteristics of conventional, Cermet and pore-filled membranes is reported in Table 10.

#### 3.5.1. Dual-Phase Ceramic-Metallic Membraners (Cermet)

**Figure 10:**
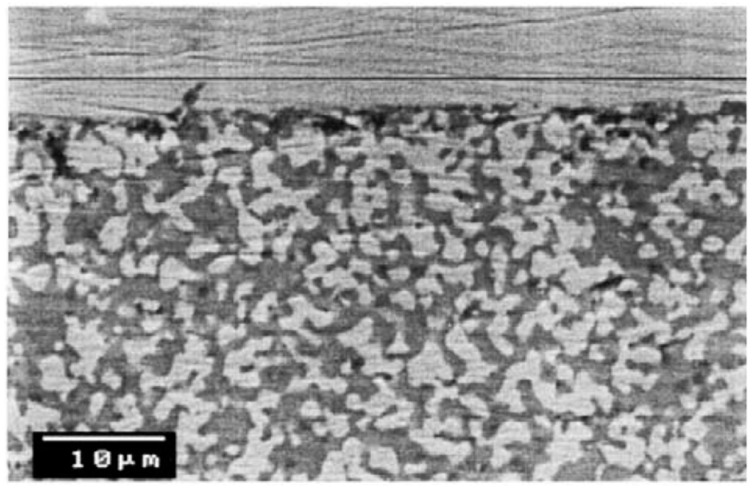
Cross section SEM image of Pd-YSZ membrane after exposure to H_2_S at 600 °C. Reprinted from [186] with permission from Elsevier.

Ceramic-metallic composite membranes or more commonly called Cermet membranes for hydrogen separation are increasingly attracting the interest of researchers. In 1994 Iwhara et al. [180] reported proton conductivity of BaCeO_3-δ_ layers and suggested the possibility to be used as hydrogen selective membranes, since these layers exhibit the highest reported proton conductivity reported so far [181]. However, their electronic conductivity is insufficient for achieving high hydrogen permeation rates without applying voltage, as reported by Balachandran et al. [182], because the measured electronic conductivity of BaCe_0.95_Y_0.05_O_3-δ_ (BCY) at 700 °C was 2.1 × 10^−3^ Ω^−1^∙m^−1^, which is 3 times smaller than the protonic conductivity. The same authors found that SrCeO_3-δ_ has a lower conductivity than the BaCeO_3-δ_ material [183]. Although the conductivity was improved by doping it with larger contents of yttrium, they found that the electronic conductivity was too low for real applications as a hydrogen separation system. Some years later, they increased the electronic conductivity of BaCe_0.8_Y_0.2_O_3-δ_ (BCY) by dispersing a metallic powder in a ceramic matrix, creating a novel metallic-ceramic matrix [184]. The authors suggested that the dispersion of metal helps the hydrogen permeability by creating an additional transport path for hydrogen. When 40% of the hydrogen transport metal was dispersed in the ceramic (BCY) matrix, the hydrogen permeation flux was enhanced. However, higher permeation rates were obtained when the perovskite structure was replaced with Al_2_O_3_ or ZrO_2_. A 40 µm thick membrane consisting of a mechanically stable ceramic mixed with a hydrogen transport metal (the authors did not specify the employed materials), exhibited a hydrogen flux of 0.108 mol∙m^−2^∙s^−1^ at 900 °C [185]. The same permeation rate was obtained with a 22 μm thick Pd (50 vol. %)-YSZ at 900 °C in the presence of pure hydrogen at atmospheric pressure [186]. The interconnected ceramic/metal networks (see Figure 10) were prepared by pressing powders into a disc-shape membrane with different thicknesses (22–200 μm). The authors observed an inverse linearity of the hydrogen flux with the membrane thickness, which suggests that bulk diffusion was the limiting step for the membrane permeation. The stability of these materials under synthetic syngas (61.3% H_2_, 8.2% CH_4_, 11.5% CO, 9% CO_2_, 0% He) and H_2_S was confirmed at 900 °C after 90 h, presenting an initial decrease in the hydrogen flux which was almost completely recovered after exposure for a couple of h to the synthetic gas with 100 ppm of H_2_S.

In 2011 Park et al. [187] employed Ta (60 vol. %) as metallic phase dispersed in YSZ since tantalum is cheaper than noble metals like Ag, Au, Pd or Pt and exhibit higher permeation rates. However, a critical point of this metal is its instability under oxygen and hydrogen containing atmospheres, which react at low temperatures with the membrane decreasing its permeation properties. Tantalum hydride was formed during permeation tests resulting in membrane failure. Before failure, the measured hydrogen flux was 8.93 × 10^−3^ mol∙m^−2^·s^−1^ at 500 °C in the presence of pure hydrogen (feed pressure 2 bar) for a membrane with a thickness of 500 μm.

In 2011 other research groups reported the preparation of cermet membranes dispersing Pd in fluorite type GDC [188] and perovskite type CaZr_0.9_Y_0.1_O_3-δ_ (CZY) [189]. Proton conducting CZY was chosen, since it is thermodynamically stable in CO_2_-containing atmospheres and mixed with electron and hydrogen conducting palladium powder. The highest hydrogen flux, 0.017 mol∙m^−2^·s^−1^, was obtained for a 500 μm thick 50 vol. % Pd-50 vol. % CZY membrane at 900 °C using a feed gas with 80% hydrogen. This value is higher than cermet membranes based on Al_2_O_3_ and YSZ as these showed lower hydrogen fluxes at the same operating conditions. The hydrogen flux for Pd/Al_2_O_3_ and Pd/YSZ membranes were 1.41 × 10^−2^ and 8.18 × 10^−3^ mol∙m^−2^∙s^−1^, respectively. In 2013 the same authors prepared a thinner (218 μm) Pd/YSZ membranes for water splitting [190], showing a higher hydrogen flux (0.024 mol∙m^−2^·s^−1^) from H_2_-H_2_O mixture with a steam partial pressure below 1 atm. Although the thermal stability is enhanced and the YSZ does not form the corresponding oxysulfide in H_2_S environment, the Pd metal reacts easily in the presence of 300 ppm H_2_S forming Pd_4_S and decreasing the hydrogen permeation flux. Luckily, when introducing steam (even as sweep gas), it reacts with Pd_4_S producing SO_2_, which “regenerates” the Pd so that the permeation flux can be recovered.

Results obtained with Pd/CZY membranes [189] where improved when the perovskite phase was replaced by GDC (Ce_0.8_Gd_0.2_O_2-δ_) [188]. GCD is also known as Gd-doped ceria or GCO (gadolinium cerium oxide). Pd/GDC operating under the same conditions as Pd/CZY presented a hydrogen flux of 0.041 mol∙m^−2^·s^−1^.

**Figure 11:**
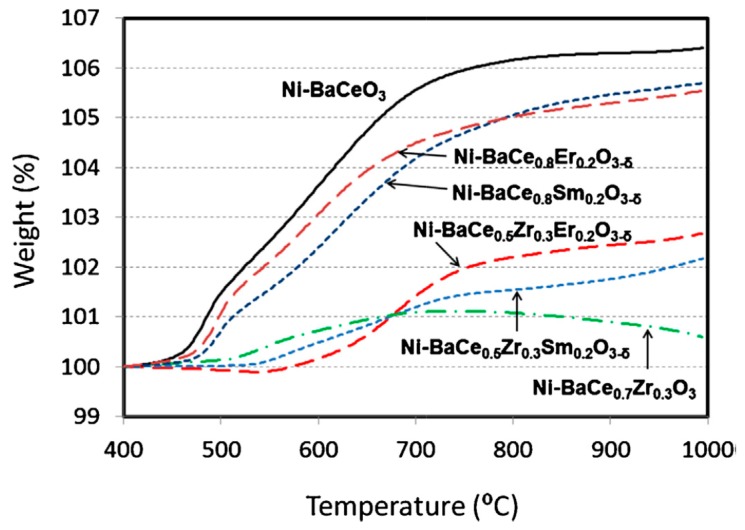
Weight change of reduced Ni-BaCeO_3_, Ni-BaCe_0.7_Zr_0.3_O_3_, Ni-BaCe_0.8_M_0.2_O_3-δ_ and Ni-BaCe_0.5_Zr_0.3_M_0.2_O_3-δ_ (M = Sm, Er) cermet powders with increasing temperature in pure CO_2_ atmosphere. Reprinted from [208] with permission from Elsevier.

As was explained before, the proton conductivity of BCY is one of the highest reported. Nevertheless, degradation of this material occurs in the presence of CO_2_ and H_2_O forming cerium oxide and barium carbonate [204], as well as BaCeO_3_ peroskite [205]. The chemical stability of yttrium-doped barium zirconate is larger than BCY, but their electron conductivity is lower due to the presence of zirconium [206]. An increase in the electron conduction rate is achieved by dispersing 40 vol. % nickel in the BZCY (Ba(Zr_0.1_Ce_0.7_Y_0.2_)O_3-δ_) phase [191,192]. A low hydrogen permeation (4.2 × 10^−4^ mol∙m^−2^∙s^−1^) was achieved at 900 °C for a 1 mm thick Ni-BZCY membrane [191]. For a thinner membrane (266 μm) the hydrogen flux was increased up to 6.0 × 10^−3^ mol∙m^−2^∙s^−1^ at 900 °C when 100% H_2_ was fed [192]. The hydrogen flux decreases in the presence of CO_2_. In particular, when using mixtures containing 10%–30% of CO_2_ and 40% of hydrogen, an initial decrease in the hydrogen flux was observed followed by a constant flux for the remaining 80 h. However, once the CO_2_ is removed from the feed gas, the initial hydrogen flux is recovered for Ni-BCZY. In contrast, unstable Ni-BCY presented a continuous decrease in the flux in the presence of 20%–30% CO_2_ [207]. The advantage of doping Ni-BCZ with rare earth metals such as erbium and samarium was reported in 2015 [208]. The chemical stability against CO_2_ was improved when erbium was used as a dopant. The chemical stability in the presence of a pure CO_2_ atmosphere of several perosvkites based on BaCeO_3_ is shown in Figure 11. The decomposition of BaCeO_3_ is promoted at higher temperatures, while substitution of Ce with samarium and erbium enhances the chemical stability. On the other hand, introduction of zirconium to BaCeO_3_, BaCe_0.8_Er_0.2_O_3-δ_ and BaCe_0.8_Sm_0.2_O_3-δ_, increases the stability for all cases, since the temperature at which barium carbonate started to be formed was increased to 550 °C. As shown in Figure 11, the chemical stability of Ni-BaCe_0.5_Zr_0.3_M_0.2_O_3-δ_ (M = Sm, Er) in the range of 550–670 °C is better than Ni-BCZ, and these cermets can be applied for hydrogen separation from methane steam reforming. 

As the hydrogen flux is increased by preparing thin membranes on asymmetric supports, Zhu et al. [193] prepared a 30 μm Ni-BZCY on top of a porous support made of BZCY(Ba(Zr_0.1_Ce_0.7_Y_0.2_)O_3-δ_). However, the obtained result was not as good as expected. At 900 °C with 80% of hydrogen in the feed gas, the permeation flux was only 2.5 × 10^−3^ mol∙s^−1^∙m^−2^, which is one order of magnitude smaller than for Pd/GDC reported by Jeon et al. [188]. Suggested reasons for the observed low flux are: (i) surface exchange becomes the rate limiting step as the thickness of the membrane decreases and (ii) too low porosity of the porous substrate. Self-supported cermet with a similar composition (50 wt. % Ni-50 wt. % BaCe_0.9_Y_0.1_O_3-δ_) was studied by Kim et al. [194], who found for a thicker membrane (230 μm) a higher hydrogen permeation flux (5.88 × 10^−3^ mol∙s^−1^∙m^−2^) than supported Ni-BZCY even at a lower temperature (800 °C) when feeding pure hydrogen. In 2013, a supported Ni-BZCYYb (BaZr_0.1_Ce_0.7_Y_0.1_Yb_0.1_O_3-δ_) was developed by Mingfei Liu et al. [195]. Incorporation of Yb enhances the ionic conductivity while maintaining the thermal and chemical stability. The highest hydrogen permeation flux, 8.33 × 10^−3^ mol∙m^−1∙^s^−1^, was measured at 900 °C when pure hydrogen and nitrogen were used on the feed and permeate side, respectively. During 2014 and 2015, many research groups investigated other types of cermets such as Ni-BZPY [209], Ni-BCTZ [210] and Ni-LCD [211], which showed stability in the presence of CO_2_ and H_2_O, while the hydrogen fluxes were below 2 × 10^−3^ mol∙m^−1∙^s^−1^ at 800 °C. 

**Figure 12:**
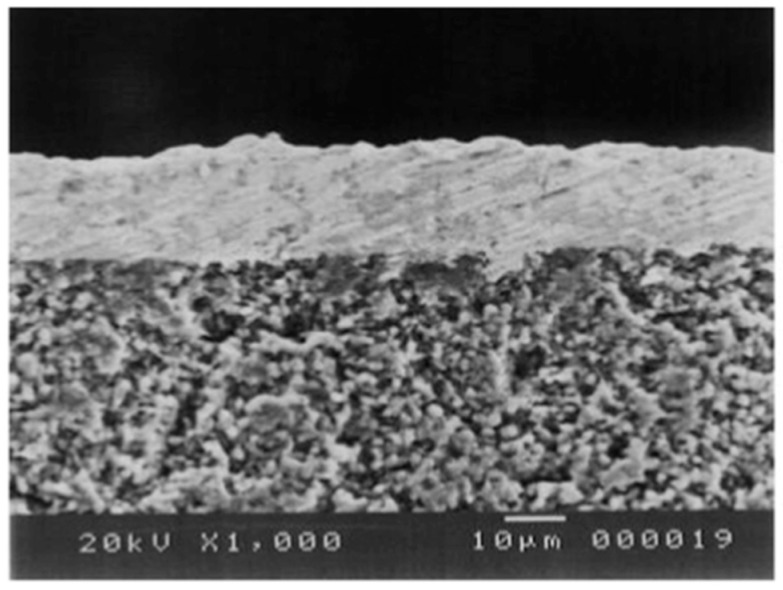
Cross section SEM image of a cermet membrane (top part) on a porous alumina support (bottom part). Reprinted from [197] with permission from Elsevier.

In 2014, a supported 18 μm thick 60 vol. % Pd/YSZ cermet membrane supported on an alumina porous substrate was reported by Balachandran et al. [197]. The dense cermet layer was supported on a porous disk made by the paste-painting method (see Figure 12). The permeated hydrogen flux increased from 0.193 mol∙m^−1∙^s^−1^ at 400 °C to 0.387 mol∙m^−1∙^s^−1^ at 900 °C for a feed gas containing 90% H_2_ (in helium) at ambient pressure and nitrogen as sweep gas. Bulk diffusion is the rate limiting step for this thin dense membrane. A long-term test was carried out for four months for a thicker membrane (25 μm) in the temperature range of 500–600 °C. The hydrogen flux was stable as the same hydrogen flux was measured at 500 °C initially and at the end of test (0.07 mol∙m^−1∙^s^−1^). Moreover, the Pd/YSZ cermet membrane was chemically stable in the presence of syngas at high pressure. The resistance to H_2_S was also determined for an even thicker membrane (200 μm) at 900 °C in the presence of 400 ppm H_2_S and 79.8% H_2_. In the first h a decrease in the hydrogen flux was observed, however, the permeated flux reached a plateau that was maintained for more than 120 h. This underlines that the Pd/YSZ cermet membrane is an interesting membrane for hydrogen production systems.

Lee et al. [212] reported a novel cermet membrane composed of Ti_4_Cr_3_Nb_3_O_2_ with 50 wt. % of PdAg. Niobium oxide was added due to the catalytic properties for ionization of hydrogen molecules. The measured permeability of this mixture was 1 × 10^−4^ mol∙m^−1∙^s^−1∙^Pa^−0.5^ at 400 °C and 300 kPa of pressure difference using a planar membrane of 1.2 mm thick.

Cermet membranes are not only applied for the separation of hydrogen from gas mixtures such as syngas, but they can also be applied in membrane reactors for other applications such as water splitting, as reported in the literature [213].

#### 3.5.2. Pore-Filled Type Membraners

The principle of hydrogen permeation in pore-filled membranes is similar to that of cermet membranes: a mixed ceramic/metal membrane is produced and used for the separation. In cermet membranes powders of both materials are mixed and pressed before sintering. However, as the name implies, in pore-fill membranes the pores of a ceramic phase are filled with the metallic phase. The employed amount of metallic phase is only a fraction in comparison with cermet membranes and much less than conventional in thin film membranes. Moreover, the deposition of a porous ceramic layer on top of a filled mesoporous layer avoids exposure of the selective material to the external environment, such as in fluidized-bed membrane reactors.

The first reported pore-filled type membrane was for oxygen perm-selective separation, as discussed by Kim et al. [214]. Before the deposition of palladium, an YSZ layer was obtained with a dip-coating method on top of an α-Al_2_O_3_ porous support, having 5 μm thick layer after being dried at 40 °C and relative humidity of 40%–50% followed by calcination at 1000 °C for 3 h. Palladium was introduced into the 100 nm pore-sized YSZ layer by a reservoir method. Palladium acetate was dissolved in a HCl-acetone mixture followed by one day drying and subsequent calcination at 500 °C for 2 h under hydrogen, in order to reduce Pd^2+^ into Pd^0^. The remaining pores were filled with YSZ by CVD employing ZrCl_4_ and YCl_3_ as metal precursors. The deposition was performed in the presence of oxygen in order to form the desired metal oxide (Y_2_O_3_-ZrO_2_) at 1000 °C. The prepared membrane was used for oxygen separation, but the presence of palladium suggested the possibility to use the same membrane as a hydrogen separation membrane.

**Figure 13:**
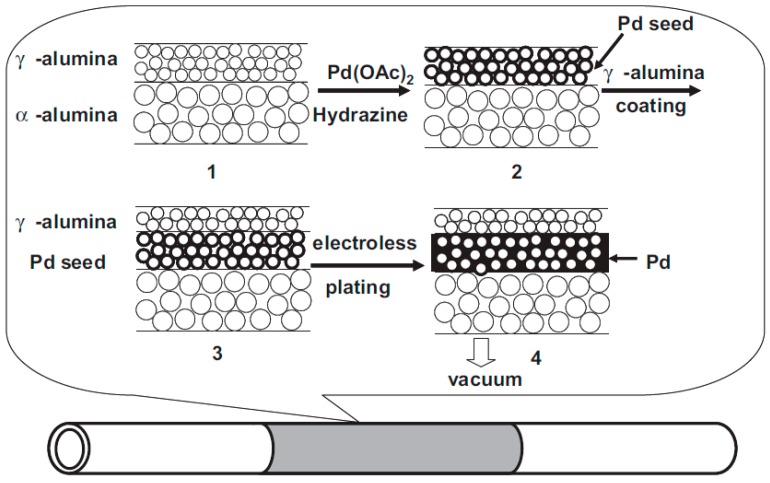
Schematic representation of pore-filled type membranes performed by Pacheco Tanaka et al. Reprinted from [111] with permission from Wiley VCH.

Pacheco Tanaka and co-workers from AIST (National Institute of Advanced Industrial Science and Technology, Tsukuba, Japan) [111] prepared pore-filled membranes for hydrogen separation introducing palladium in a thin nanoporous γ-Al_2_O_3_ layer deposited on top of a tubular porous α-alumina support by vacuum-assisted dip-coating of a boehmite sol containing PVA and PEG. The calcination was performed at 600 °C for 3 h. Palladium seeds were introduced in the 5 μm thick γ-Al_2_O_3_ layer by dipping into a palladium acetate dissolved in chloroform followed by reduction (Pd^2+^ → Pd°) in hydrazine. Afterward, a ceramic protective layer (γ-Al_2_O_3_) was deposited on top of the previous Pd-activated ceramic layer using the same approach. During the cool-down step after calcination, a hydrogen stream was introduced to reduce possible oxidized palladium. Finally, the activated Pd seeds were grown by vacuum-assisted ELP. A schematic representation of this membrane configuration is represented in Figure 13. Reduction of palladium grains to nm size suppresses α-β transition and the membrane can work at lower temperatures [213,214]. Indeed, these membranes were tested in the temperature range from 50 to 300 °C without failure of the membrane and the best results were obtained at 300 °C and a pressure difference of 400 kPa, yielding a hydrogen flux of ~0.55 mol∙m^−2^∙s^−1^ and a H_2_/N_2_ perm-selectivity of 1000 [111]. Due to the difference in thermal expansion coefficient between alumina and Pd (Table 4) the permeation test at temperatures above 400 °C resulted in membrane failure. To avoid this problem, the mesoporous γ-Al_2_O_3_ layer was replaced by an YSZ/γ-Al_2_O_3_ layer in order to minimize the difference in thermal expansion with the metal [117]. The effect of the amount of YSZ was studied and it was observed that the nitrogen flux was increased when the amount of YSZ was increased. The measured hydrogen flux at 500 °C and a pressure difference of 400 kPa was ~0.616 mol∙m^−2^∙s^−1^, which was half of the flux achieved for a conventional supported thin film with the same membrane thickness (3.23 μm). The ideal H_2_/N_2_ perm-selectivity was 350. Since the amount of palladium is only a fraction of that employed for films and YSZ-Al_2_O_3_ does not transport hydrogen, the obtained results are as expected. Long-term tests were performed for 200 h in the temperature range of 500–600 °C. A gradual loss in the hydrogen flux was observed during the first 100 h at 500 °C, then the temperature was increased to 600 °C, showing an initial hydrogen flux increases related to the temperature effect. Again, the hydrogen flux decreased during the first 20 h at 600 °C. Afterward a stable permeated hydrogen flux was maintained for longer times. The preparation of this type of membranes was patented in 2006 [215].

**Figure 14:**
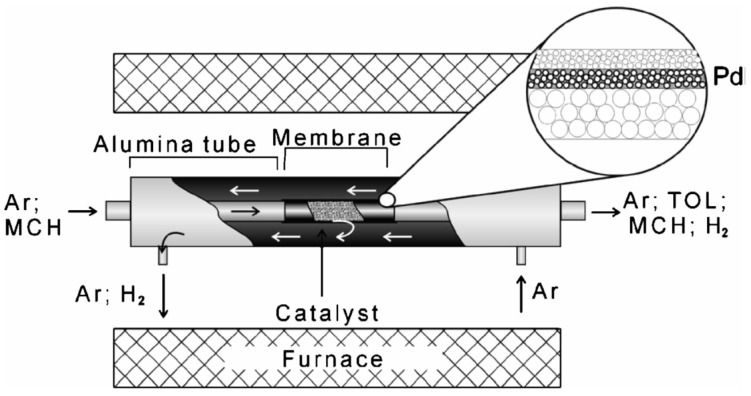
Schematic representation of a pore-filled type membrane integrated in a packed-bed membrane reactor for the dehydrogenation of methylcyclohexane into toluene and hydrogen. Reprinted from [213] with permission from The Chemical Society of Japan.

A pore-filled type membrane was integrated in a membrane reactor for hydrogen production by dehydrogenation of methylcyclohexane producing toluene and hydrogen [213]. Platinum supported onto alumina particles was used as catalyst, mixed with sand and introduced into the inner part of the tube, where the palladium was located (see Figure 14). The reaction was performed in the temperature range below the critical temperature for α-β phase transition, 150–325 °C without formation of coke onto the catalyst bed. The introduction of the membrane into the reactor improved the conversion of the reaction to 60%–80% in a temperature range of 150–225 °C, while at higher temperatures complete conversion in both systems was achieved, where the effect of the membrane was only visible in the recovery of the hydrogen, which increased as the temperature increased. The stability of membrane was proven for 600 h confirming constant membrane efficiency (i.e., flux and selectivity).

The use of YSZ guarantees better thermal properties than palladium membranes as was reported for a pore-fill-type membrane prepared with an YSZ porous support and coated with a mesoporous layer of the same material [214]. The hydrogen selectivity was improved by adding a surfactant (Triton^®^ X-100) to the plating solution in order to decrease the surface tension. The hydrogen flux at 600 °C and 50 kPa of pressure difference increased from 0.956 mol∙m^−2^∙s^−1^ (without addition of the surfactant) to 1.368 mol∙m^−2^∙s^−1^ when the concentration of the surfactant in the resulting plating solution was 0.05 wt. %. The H_2_/N_2_ ideal perm-selectivity for a membrane prepared without addition of the surfactant was 1200, while for the membrane with 0.05 wt. % of surfactant in the plating solution the resulting ideal perm-selectivity was 20,000.

Some years later (2012) the AIST research group reported a study where hydrogen permeation of thin-film and pore-filled type membranes were compared in the presence of steam and CO_2_ [198]. A thin palladium membrane was deposited by chemical vapor deposition onto an α-Al_2_O_3_ porous support, while pore-filled type membranes were produced in a similar way as reported earlier [111]. The thickness of the deposited film by CVD was around 1–2 μm, while the embedded Pd on γ-Al_2_O_3_ layer was close to 10 μm thick. The filled pores presented a distribution in pore size of about 1–9 nm and a surface area of 213 m^2^∙g^−1^ after calcination at 600 °C, which means that the Pd particle size was in the nano-range. In the presence of pure hydrogen, the permeance of the conventional thin-film membrane was a factor three larger than the pore-filled type membrane, 1.3 × 10^−3^ mol∙m^−2^∙s^−1^∙Pa^−0.5^ for the thin film and 4.1 × 10^−4^ mol∙m^−2^∙s^−1∙^Pa^−0.5^ for the pore-filled membrane at 300 °C and a pressure difference of 200 kPa. The hydrogen permeation rates decreased for both membranes when CO_2_ was introduced into the gas stream at 300 °C. However, the CO_2_ poisoning was more pronounced for the conventional thin-film membrane than for pore-filled membrane. Moreover, the hydrogen flux was completely recovered when CO_2_ was removed for the pore-filled membrane, whereas only 80% of the initial hydrogen flux was recovered for the CVD membrane. The introduction of steam decreases the initial hydrogen flux for both membrane types, but the flux was successfully recovered after exposure to steam for both membranes. Concerning the perm-selectivity, for the conventional membrane the initial H_2_/N_2_ separation factor was ~883 (re-calculated values explained at Table 10), which was decreased to ~447 and ~409 after exposure to CO_2_ and steam, respectively. For the pore-filled membrane, the hydrogen flux before and after exposure to CO_2_ and H_2_O remain the same, ~0.095 mol∙m^−2^∙s^−1^ at 200 kPa (4.75 × 10^−7^ mol∙m^−2^∙s^−1^∙Pa^−1^) and an initial selectivity of ~176. No changes were reported by the authors for the pore-filled membrane. However, re-calculated selectivities after exposure to CO_2_ and steam were ~206 and ~12, respectively.

**Figure 15:**
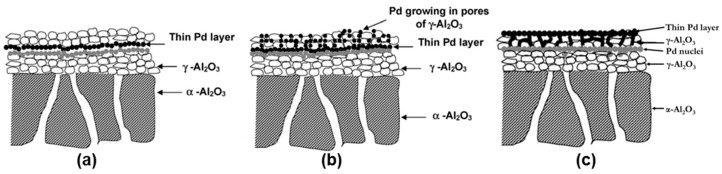
Schematic representation of encapsulated Pd (**a**), Pd encapsulated nanopore (**b**) and Pd nanopore (**c**). Reprinted from [199] with permission from Elsevier.

A similar concept was reported by Nair et al. [199] for a hollow fiber configuration. Three types of configurations were reported, the so-called “encapsulated Pd (M-E)”, “Pd encapsulated nanopore (M-EN)” and “Pd nanopore (M-N)” membranes. All these types of membranes were supported on an α-Al_2_O_3_ hollow fiber followed by boehmite slip casting. For the Pd encapsulated case, the γ-alumina surface was activated with Pd followed by growth of a thin Pd layer by ELP. Subsequently, the surface was coated with another γ-alumina layer (Figure 15a). The second type of membrane, “Pd encapsulated nanopore”, can be easily prepared by filling the resulted pores of the “encapsulated Pd membrane” with palladium using ELP, connected with the first deposited Pd layer as shown in Figure 15b. The equivalent palladium thickness was 1.6 μm, but the amount of deposited palladium was relatively low in comparison with convectional supported membranes.

The other membrane configuration, “Pd nanopore” (Figure 15c), differs from the other two configurations in that not only pores are filled, but also a 2 μm thick Pd layer is grown on top of the alumina layer. Permeation tests were performed at 370 °C and a feed side pressure of 300 kPa, observing a higher hydrogen flux and perm-selectivity for the encapsulated nanopore membranes. Indeed, the measured hydrogen flux (and selectivity) for Pd-encapsulated nanopore, Pd encapsulate and Pd-nanopore membranes were ~0.25 mol∙m^−2^∙s^−1^ (H_2_/N_2_ = 8750), ~0.26 mol∙m^−2^∙s^−1^ (H_2_/N_2_ = 3800), and ~0.13 mol∙m^−2^∙s^−1^ (H_2_/N_2_ = 550), respectively. Thermal stability was confirmed at the same temperature (370 °C) for 150–200 h, resulting in an increase of the hydrogen flux at lower pressures for the encapsulated nanopore membrane, 0.67 mol∙m^−2^∙s^−1^, while the perm-selectivity decreased to 160. The selectivity of the nanopore membrane was reduced to half of the calculated value before thermal aging, while the hydrogen flux remained close to 0.1 mol∙m^−2^∙s^−1^. The largest decrease in terms of selectivity was for the encapsulated membrane, 10 times lower (380) with a hydrogen flux increase from 0.26 to 0.5 mol∙m^−2^∙s^−1^. The low selectivity of these membranes was associated to thermal stress caused by the discontinuities between alumina and palladium due to differences in thermal expansion, as for the pore-filled membranes prepared by Pacheco Tanaka and coworkers [111]. The same group also showed similar results for a nanopore type membrane [200], where the hydrogen flux and ideal perm-selectivity at 500 °C and a pressure difference of 4 bar were 0.56 mol∙m^−2^∙s^−1^ and 6600, respectively. On the other hand, Arratibel et al. [201] recently reported a pore-filled membrane with a high stability at 500 °C and 550 °C after 900 h at 50 kPa of pressure difference. Palladium seeds were deposited into the nanoporous layer of YSZ/γ-Al_2_O_3_. However, the hydrogen flux (9.04 × 10^−3^ mol∙m^−2^∙s^−1^) and perm-selectivity (~50) were too low at the end of the experiment at 550 °C.

Another way to prepare pore-filled type membranes was patented in 2009 by Wakuy et al. [215]. The authors first deposited a thin mesoporous ceramic layer followed by activation of the surface by filling the pores with Pd particles. Afterward, the deposited ceramic layer with palladium seeds was removed leaving a portion of the deposited Pd into the original porous surface. Again, a ceramic layer was deposited as a protective layer followed by electroless plating to grow palladium and completely fill the pores.

Ohio State University patented in 2010 a pore-filled type membrane employing ZrO_2_ as an intermediate layer between the support and palladium layer [202]. They suggested deposition of ZrO_2_/Pd nano-composite on top of a macroporous support, consisting of Al_2_O_3_ or ZrO_2_, followed by deposition of a thin Pd (or Pd alloy) layer by ELP with a thickness ranging from 10 nm to 1 μm. The ZrO_2_/Pd (30/70 vol. %) and the macroporous layers were also deposited on top of the Pd or Pd-alloy layer to protect the hydrogen selective metals. There is no chemical adhesion between Pd and ZrO_2_, but the adherence of ZrO_2_ with the macroporous layer is easier due to their chemical affinity. A membrane supported on top of γ-alumina with a 120 nm Pd/ZrO_2_ composite layer was covered with 215 nm Pd and tested in the presence of pure hydrogen at 200, 260 and 320 °C. The measured fluxes were 0.04, 0.1 and 0.25 mol∙m^−2^∙s^−1^ respectively, with a pressure difference of 2 bar across the feed and permeate sides.

The most recent reported study on pore-filled type membranes was performed by Yogo and co-workers [203]. They deposited directly Pd into α-Al_2_O_3_ porous tubes by dip-coating with an average pore size of 100 nm using a Pd-gel containing PdCl_2_ as palladium carrier in HCl and agarose L polymer as binder. An ice bath was used to cool the Pd-gel onto the porous support followed by reduction in the presence of hydrazine. After cleaning, 10 min of electroless plating was performed in order to grow the Pd seeds. Before carrying out a loner ELP, the Pd-gel was removed by calcination of the sample at 850 °C. The palladium layer obtained inside the porous support was around 8–10 μm at 10 μm depth. The amount of palladium used for the pore-filled membranes was 34% of that required for a supported thin-film membrane with the same thickness. The hydrogen permeance at 500 °C and 150 kPa of pressure difference was 1.2 × 10^−6^ mol∙m^−2^∙s^−1^∙Pa^−1^, and stable for 150 h at different temperatures (200–500 °C) in the presence of a gas mixture H_2_/N_2_. The ideal perm-selectivity of this membrane was above 10,000. In conclusion, pore-filled type membranes represent an interesting alternative when membranes are integrated in a fluidized bed membrane reactor to avoid abrasion problems of the membrane surface due to the scouring action of the fluidized catalyst particles. 

## 4. Conclusions

In this review, an overview of the latest achievements with different types of membrane reactors with different types of integrated H_2_ perm-selective membranes for the production of H_2_ from different feedstocks has been given. Requirements that need to be fulfilled by H_2_ perm-selective membranes for its integration in packed or fluidized bed membrane reactors are discussed, as well as the selection of the materials for the support and interdiffusion layer. Depending on the operation conditions, stainless steel (316 L) and ZrO_2_/YSZ as a support and diffusion barrier are preferred for applications at around 400–500 °C. For applications at higher operating temperatures supports with higher stability are required, such as Hastelloy X and Ni-based alloys (Inconel). 

Concerning the material for the selective layer, Pd-based membranes represent the best choice in comparison with non-palladium alloys (V, Nb, Ta), yielding higher permeabilities than pure Pd. However, their tendency to embrittlement is also high. PdAg membranes exhibit a high flux, PdAu membranes a high sulfur resistance, whereas PdAgAu membranes combine both properties. However, for high-temperature applications such as methane steam reforming, alloys like PdRu and PdPt are requited for improved thermal stability at temperatures above 500 °C. Combination of all these elements to create a membrane which could work under extreme conditions is an interesting option. Nevertheless, the possible techniques for their preparation is limited to PVD, due to the complexity to prepare these mixed membranes with ELP. 

Integration of supported membranes in fluidized bed membrane reactors require membranes with improved abrasion resistance because of the scouring action of the catalyst particles which may deteriorate the membrane properties dramatically. Cermet membranes may represent an interesting option, since part of their surface do not suffer from erosion since they are much harder than the catalyst particles. However, the hydrogen selective material is not completely covered. For this reason, pore-filled membranes are suggested as the best alternative to avoid membrane abrasion. They have been investigated for their application in the dehydrogenation of methylcyclohexane at low temperatures with a high stability during 600 h. However, their use in membrane reactors for hydrogen production via reforming of hydrocarbons (methane, methanol or ethanol) is very interesting, but has not been studied yet.

## Figures and Tables

**Table 1:** Reactions involved in hydrogen production from methane, methanol and ethanol, and their enthalpies [35,36,37,38,39,40,41].

Reaction	$\Delta H_{298K}\;\left(kJ\cdot mol^{-1}\right)$
**Water gas shift (WGS)**	
CO + H_2_O = CO + H_2_	−41.1
**Decomposition (Carbon production)**	
CH_4_ = C + 2H_2_	75
**Steam reforming (SR) reactions**	
CH_4_ + H_2_O = CO + 3H_2_	206.2
CH_4_ + 2H_2_O = CO_2_ + 4H_2_	164.9
CH_3_OH + H_2_O = CO_2_ + 3H_2_	49
C_2_H_5_OH + H_2_O = 2CO + 4H_2_	239.5
**Partial and full oxidation reactions**	
CH_4_ + 2O_2_ = CO_2_ + 2H_2_O	−14.4
CH_4_ + O_2_ = CO_2_ + 2H_2_	−71
CH_4_ + ½ O_2_ = CO_2_ + 2H_2_	−35.6
CH_3_OH + ½ O_2_ = CO_2_ + 2H_2_	−192.3
C_2_H_5_OH + ½ O_2_ = 2CO + 3H_2_	−14.4
**Autothermal reforming (ATR) reactions**	339
4CH_4_ + 2H_2_O + O_2_ = 10H_2_ + 4CO	0
4CH_3_OH + 3H_2_O + ½ O_2_ = 4CO_2_ + 11H_2_	−50
C_2_H_5_OH + 2H_2_O + 3/2 O_2_ = 2CO_2_ + 5H_2_	

**Table 2:** Performance and operating conditions of several membrane reactors for reforming of methane, methanol and ethanol, reported in the last years.

Membrane	Method	MR Type	Catalysts	Reaction Type ^a^	T (°C)	ΔP (kPa)	Feedstock	H_2_O/Carbon	O_2_/Feedstock	GHSV (h^−1^)	Conversion (%)		H_2_ Recovery (%)	H_2_ Purity (%)	Reference
											Equilibrium	MR			
PdAg (4–5 μm)/Al_2_O_3_	ELP	PB	Pt_3_Ni_10_/CeO_2_	SR	525	1000	CH_4_	3	-	136	65	90	80	-	[51]
Pd/V/Pd (1/100/1 µm)	PVD-MS	PB	Ni/SiO_2_	ATR	400	-	CH_4_	1.25 ^e^	0.3 ^e^	-	26	~45	-	-	[52]
Pd (3 μm)/Al_2_O_3_(HF)	ELP	PB	Ni/SBA-15	SR	560	-	CH_4_	2	-	-	52	53	43	-	[53]
Pd (10.2 µm)/OxPSS	ELP	PB	Fe-Cr oxide	SR	400	2–3	CH_4_	1 (H_2_O/CO)	-	5000	32	59	15	-	[54]
Pd/Nao/PSS	ELP	PB	Ru/La_2_O_2_CO_3_	CR	450	-	CH_4_	-	0.3	-	35	36	80	99.5	[55]
PdAg	-	PB	Ni/γ-Al_2_O_3_	SR	600	100	CH_4_	2.85 (CH_4_/CO_2_)	-	-	20	35	47	-	[56]
Pd/PSS	-	FB	-	SR	450	1000	CH_4_	3 (H_2_O/CO)	-	-	-	-	47	-	[57]
PdAg (25 μm)	-	FB	-	ATR	600	2600	CH_4_	2–3.5	0.3	-	29	41	-	-	[58]
PdAg (25 μm)/PSS	-	FB	NiO/Al_2_O_3_	SR	550	900	CH_4_	3	-	-	-	73.1	-	99.94	[59]
PdAg (25 μm)/PSS	-	FB	NM ^b^/Al_2_O_3_	ATR	550	900	CH_4_	3	0.35	-	-	80.9	-	99.988	-
PdRu (5 μm)YSZ/PSS	ELP	FB	Ni	SR	580	2900	CH_4_	3	-	150	17	90	85	95.59	[60]
PdAg/Inconel/PdAg	ELP	FB	Noble metal-based	SR	550–630	350–550	CH_4_	3	-	0.26 ^h^	72.2	83.1	-	~100	[61]
PdAg/YSZ-Al_2_O_3_/HastelloyX	ELP	FB	Ni/CaAl_2_O_3_	SR	600	300	CH_4_	3	-	-	61	73	27	97.6	[62]
PdAg/YSZ-Al_2_O_3_/HastelloyX	ELP	FB	Ni/CaAl_2_O_3_	ATR	600	300	CH_4_	3	0.25	-	72	82	34	97.1	[62]
PdAg/Al_2_O_3_	ELP	FB	Ru/Ce_0.75_Zr_0.25_O_2_	SR	600	130	CH_4_	3	-	-	89	89.3	23	99.98 ^g^	[63]
PdAg/Al_2_O_3_	ELP	FB	Ru/Ce_0.75_Zr_0.25_O_2_	ATR	600	130	CH_4_	3	0.25	-	93.2	96.7	35	99.95 ^g^	[63]
Pd (7 μm)/Al_2_O_3_	ELP	PB	Cu-Zn/Al_2_O_3_	SR	330	250	CH_3_OH	2.5	-	18500	-	85	40	~100	[64]
PdAg (50 μm)	Cold-work	PB	Cu-Zn/Al_2_O_3_	SR	280	250	CH_3_OH	3	-	1800	91	100	~46	~100	[22]
Pd (7 μm)/Al_2_O_3_	ELP	PB	CuO/ZnO	SR	300	150	CH_3_OH	1.5	-	0.95 ^h^	-	97	72	~91	[65]
PdAg (5 μm)/Al_2_O_3_(HF)	ELP	PB	Cu-Zn/GaOx ^c^	SR	400	100	CH_3_OH	2	-	-	88	100	~50	-	[66]
Pd (24.3 μm)/PSS		PB	Ni-Zn/Al_2_O_3_	SR	200–300	345	CH_3_OH	1	-	-	-	15–78	~30–50	-	[67]
PdRu	-	PB	Pt-Ru	SR	450	-	C_2_H_5_OH	4.5	-	-	-	98–99	35–60	99.99	[40]
PdAg (75 μm)	-	PB	Rh/CeO_2_	SR	550	-	C_2_H_5_OH	5	-	-	-	100	70	-	[68]
PdAg (30 μm)	-	PB	Co[Si_2_O_5_](OH)2 ^f^	SR	350	1400	C_2_H_5_OH	3	-	-	-	100	80	-	[69]
PdAg (50 μm)/PSS	-	PB	Co/Al_2_O_3_	SR	400	150	C_2_H_5_OH	9.35	-	5.5 ^h^	84	94.2–94.5	27	~100	[70]
PdAg (50 μm)/PSS	-	PB	Co/Al_2_O_3_	SR	400	300	C_2_H_5_OH	9.35	-	-	--	100	95	~100	[71]
PdAg (50 μm)/PSS	-	PB	Co hydrocalcyte ^f^	SR	600	1200	C_2_H_5_OH	1.5	-	-	-	100	85	-	[72]
Pd (20 μm)/PSS	ELP	PB	Co/Al_2_O_3_	SR	400	1200	C_2_H_5_OH	2	-	-	-	94	~40	95	[73]
Pd(4–5 μm)/Al_2_O_3_	ELP	PB	Pt-Ni/CeO_2_-SiC foam	SR	340–480	400–800	C_2_H_5_OH	1.5	-	-	-	100	70	>99.5	[74]
Pd (8 μm)/Al_2_O_3_	ELP	PB	Ni/CeO_2_	SR	400	300	C_2_H_5_OH^g^	6.5	-	5000	-	98	70	80	[75]
PdAg (60 μm)	Cold work	PB	Pt-based/Al_2_O_3_	ATR	450	200	C_2_H_5_OH	5	0.5	-	-	-	4.01 ^d^	-	[76]
PdCu (2 μm)/γ-Al_2_O_3_	ELP	PB	Na-Co/ZnO	SR	350	100	C_2_H_5_OH	1.5	-	-	50	62	-	99.8	[77]
Pd (1.3 μm)/Al_2_O_3_(HF)	ELP	PB	Na-Co/ZnO	SR	360	100	C_2_H_5_OH	6.5	-	9800	53	74	-	-	[78]
PdCu (2 μm)/Al_2_O_3_(HF)	ELP	PB	Na-Co/ZnO	SR	360	100	C_2_H_5_OH	6.5	-	9800	53	58	-	-	[78]
PdAg/YSZ (HF)	ELP	PB	NiO/MgO-CeO_2_	SR	350	100	C_2_H_5_OH	2.5	-	-	-	-	71	~100	[79]
PgAg (30 µm)/Inconel	-	PB	Pd-Rh/CeO_2_^f^	SR	650	1100	C_2_H_5_OH	3	-	-	-	100	54	-	[80]
PdAg (30 µm)/PSS	-	PB	Pd-Rh/CeO_2_^f^	SR	600	1200	C_2_H_5_OH	1.6	-	60–140	-	100	90	-	[81]

^a^ SR: Steam reforming; ATR: Autothermal reforming; ^b^ NM: Noble metals; ^c^ Catalysts inside pores of hollow fibres; ^d^ Hydrogen yield (produced H_2_ mol per ethanol mol fed); ^e^ Volume ratio; ^f^ Over cordierite; ^g^ Calculated purities knowing the amount of impurities. ^h^ WHSV: weight hourly space velocity: methane feed flow in kg/h. relative to catalyst load in kg/h.

**Table 3:** US DOE 2015 target for hydrogen separation membranes [6].

Performance Criteria	2015 Target
Flux	1.135 mol∙m^−2^∙s^−1^ (300 Scfh∙ft^−2^)
Membrane Cost	<5400 $∙m^−2^ (< 500 $∙ft^−2^)
Durability	>5 years
H_2_ recovery	90%
Hydrogen purity	99.99%

**Table 4:** Thermal expansion coefficients of some materials used for the preparation of hydrogen selective membranes [105,106,107].

Material	Thermal Expansion Coefficient (10^−6^ °C^−1^)
Al_2_O_3_	6.5–7.6
YSZ	10
ZrO_2_	10
CeO_2_	11.0
TiO_2_	9.2
316L	16.0
Pd	11.8
Ag	18.9–19.7
Au	14.2
Cu	16.5–17.0
Ni	11.8–13.3

**Table 5:** Required Pd thickness to cover supports with different pore sizes [109].

Media Grade of Support (μm)	Thickness of Deposited Pd Layer (μm)
5 × 10^−3^	0.8
0.1	2.2
0.2	4.5
0.3	13

**Table 6:** Comparison of the hydrogen permeation for various composite membranes.

Membrane/Support	Thickness (µm)	Permeance (mol∙m^−2^∙s^−1^∙Pa^−1^)	Temperature (°C)	Selectivity H_2_/N_2_	Reference
Pd-Ag/γ-Al_2_O_3_/α-Al_2_O_3_	0.117	7.7 × 10^−8^	300	~3800 *	[110]
Pd_95_Cu_5_/ZrO_2_/α-Al_2_O_3_	1.3	4.5 × 10^−6^	365	127	[112]
Pd-Ag/SiO_2_/Al_2_O_3_	0.15	1.4 × 10^−6^	300	600–900	[113]
Pd/γ-Al_2_O_3_/α-Al_2_O_3_ (HF)	2–3	1.1 × 10^−6^	400	>1000	[114]
Pd/TiO_2_/α-Al_2_O_3_	0.1	3.3 × 10^−6^	450	4.7	[115]
Pd/TiO_2_/Al_2_O_3_-ZrO_2_	0.4	4.8 × 10^−6^	430	83–130	[116]

* H_2_/He selectivity.

**Table 7:** Recently reported studies employing porous metal supports: materials and suppliers.

Material	Supplier	Media Grade (µm) *	Pore Size (µm)	Reference
Stainless steel	Mott Metallurgical Corporation	0.1	2–5	[54,125]
		0.2	10	[122,123]
		0.5	20	[108]
	Pall Corporation	0.1	2	[126]
	Pall Corporation (30 µm YSZ)	-	-	[127,128,129]
	GKN Sinter Metal **	-	-	[130]
Nickel	Vale Inco Pacific Ltd. **	-	-	[106,131]
Hastelloy X	Mott Corporation	0.2	-	[62,132]

* Particle retention size. 95% rejection of particles with a size greater than the grade is guaranteed. ** Powder provided by GKN and Vale Inco. Authors manufactured the porous supports.

**Table 8:** Pd-based layers deposited onto modified metallic supports with different interdiffusion barriers.

Interdiffusion Barrier		Selective Layer				Membrane Performance					Reference
Material	Deposition Method	Thickness (µm)	Material	Method	Thickness (µm)	T (°C)	Pressure Difference (kPa)	n	H_2_ Permeance (mol∙m^−2^∙s^−1^∙Pa^−n^)	H_2_/N_2_ Selectivity	
Barriers deposited onto porous stainless steel supports											
γ-Al_2_O_3_	Dip coating Sol-gel	3–4	Pd	ELP	11	-	-	-	-	-	[138]
Al_2_O_3_	2 step Dip coating	~3	Pd	ELP	5	550	340	0.5	3.39 × 10^−3^	-	[139]
Al_2_O_3_	2 step Dip coating	-	Pd	ELP	4.4	500	800	0.5	2.94 × 10^−3^	1124 *	[140]
Al_2_O_3_	Vacuum assisted Dip-coating	<1	Pd_92_Au_8_	ELP	24						[123]
ZrO_2_	PVD-MS	2.0	Pd	ELP	14	500	50	0.5	8.93 × 10^−4^	~180	[120]
ZrO_2_	PVD-MS **	0.5									[142]
ZrO_2_	Vacuum assisted dip-coating	<<1	Pd_92_Au_8_	ELP	12	450	100	0.5	1.26 × 10^−3^	>10,000	[123]
ZrO_2_	Vacuum assisted dip-coating	~5	Pd	ELP	7.5	600	500	0.5	2.53 × 10^−3^	685	[108]
ZrO_2_	Vacuum assisted dip-coating	-	Pd_78_Ag_9_Au_13_	ELP	~14	450	50	0.5	1.15 × 10^−3^	-	[124]
ZrO_2_	Vacuum assisted dip-coating	-	Pd_90_Ag_10_	ELP	~14	450	50	0.5	1.46 × 10^−3^	-	[124]
ZrO_2_	Coating by sucking	-	Pd	ELP	10	550	100	0.5	6.86 × 10^−4^	-	[143]
Pd-ZrO_2_	Dip coating	-	Pd_84_Cu_16_	ELP	5	480	250	0.5	2.19 × 10^−3^	-	[144]
YSZ	-	~30	Pd_77_Au_23_	ELP	5.7	400	~590	0.5	2.19 × 10^−3^	690	[127]
YSZ	-	~30	Pd_67_Ag_13_Au_20_	ELP	9.3	400	~590	0.5	1.44 × 10^−3^	2200	[127]
YSZ	-	~50	Pd_95_Au_5_	ELP	2.3	400	138 (Feed)	0.6	2.72 × 10^−3^	82,000	[128]
YSZ	-	~30	Pd	ELP	4.9	550	50	0.5	2.39 × 10^−3^	1750	[129]
YSZ	-	~30	Pd_73_Pt_27_	ELP	4.4	550	50	0.5	8.82 × 10^−4^	626	[129]
YSZ	-	~30	Pd_99.7_Ru_0.3_	ELP	6	550	50	0.5	2.1 × 10^−3^	1860	[129]
YSZ	APS	~100	Pd	ELP	13.6	400	250	0.5	2.88 × 10^−4^	∞	[125]
YSZ	APS	80–100	Pd	ELP	25.3	600	200	0.5	5.7 × 10^−4^	~225	[130]
YSZ	APS	10–70	Pd	ELP	23	500	50	0.5	8.43 × 10^−4^	~200	[120]
YSZ	Dip coating	2.5–3	Pd	ELP	11	650	~220	0.5	1.05 × 10^−3^	-	[145]
Cr_2_O_3_	Oxidation in air at 800 °C, 12 h	-	Pd_90.2_Ag_3.6_Cu_6.2_	ELP	40	280	90	0.5	4.32 × 10^−4^	700	[122]
Cr_2_O_3_	Oxidation at 650 °C, 12 h	-	Pd	ELP	10.2	400	250	0.5	3.3 × 10^−4^	∞	[54]
Cr_2_O_3_	Oxidation at 800 °C	-	Pd	ELP	25	600	~220	0.5	1 × 10^−4^	-	[145]
Cr_2_O_3_	Oxidation at 700 °C, 6 h and electrodeposition	2–10	Pd	ELP	32	500	100	0.5	5.84 × 10^−5^	-	[147]
TiO_2_	WPS	<15	Pd	ELP	14.4	600	200	0.5	2.6 × 10^−3^	~160	[130]
TiO_2_	WPS	40–60	Pd	ELP	9	500	50	0.5	1.91 × 10^−3^	~800	[120]
CeO_2_	Suction of Ce(OH)_4_ sol	-	Pd	ELP	10	500	100	0.5	1.36 × 10^−3^	108 **	[146]
CeO_2_	Suction of Ce(OH)_4_ sol	-	Pd	ELP + CVD	6.4	500	100	0.5	1.79 × 10^−3^	565 **	[146]
Barriers deposited onto porous nickel supports											
Al_2_O_3_	Sputtering	~0.2	Pd_97_Au_3_	PVD	-	-	-	-	-	-	[131]
ZrO_2_	Sputtering	~0.2	Pd_97_Au_3_	PVD	3.05	450	2000	0.83	1.52 × 10^−6^	2000	[131]
CeO_2_	Dip-coating	~0.5	Pd_93_Cu_7_	PVD	7	500	400	0.5	7 × 10^−4^	50,000	[106]
Al_2_O_3_-YSZ	Dip-coating	1	PdAg	ELP	4–5	400	100	0.5	7.69 × 10^−4^	200,000	[132]

* H_2_/He selectivity; ** H_2_/Ar selectivity.

**Table 9:** Characteristics of Pd/H phases and pure Pd [147,148].

Phase	Pd/H	Lattice Parameter (Å)
α	≤1/0.015	3.889–3.894
β	≥1/0.6	≥4.025
Pd-metal	-	3.889

**Table 10:** Permeation data of some conventional supported membranes, cermet membranes and pore-filled membranes.

Membrane Materials		Preparation Method	Selective Layer Thickness (μm)	T (°C)	H_2_ Flux (mol∙m^−2^∙s^−1^)	Pressure Difference (kPa)	n	Permeance H_2_ (mol∙m^−2^∙s^−1^∙Pa^−n^)	H_2_/N_2_ Selectivity	Reference
Porous Support/Inter-Diffusion Layer	Selective Layer									
PSS	Pd_77_Ag_23_ (+TT air)	PVD-MS	2.8	400	18.43	2500	0.5	1.46 × 10^−2^	2900	[126]
α-Al_2_O_3_	Pd_(90–96)_Au_(4–10)_	ELP	~4.5	600	~0.8	400	0.5	~2.0 × 10^−3^	1000	[150]
α-Al_2_O_3_	Pd_79_Ag_16_Au_5_	ELP	~4	300	~0.17	100	0.5	1.12 × 10^−6^	~8800	[159]
Self-supported	V_0.9_Fe_0.05_Al_0.05_	Arc melting	400 ^a^	400	0.170	700	0.5	3 × 10^−4^	-	[175]
Self-supported	Pd-V-Pd	ELP	2-100-2	400	1.12	600	0.5	1.8 × 10^−3^	∞	[174]
PSS	(Ni_0.6_Nb_0.4_)_0.7_Zr_0.3_	Flow casting	50–90 ^b^	450	0.155	627	0.5	1.4 × 10^−8^	-	[178]
Self-supported	60 vol. %. Ta-YSZ	Uniaxial Pressed	500	500	8.93 × 10^−3^	<200	-	-	-	[187]
Self-supported	50 vol. % Pd-GDC	Isostatically pressed	282	900	0.041	<100	0.5	1.74 × 10^−4^	-	[188]
Self-supported	50 vol. % Pd-CZY	Pressed	500	900	0.017	<100	0.5	6.2 × 10^−5^	-	[189]
Self-supported	50 vol. % Pd-YSZ	Isostatically pressed	218	900	0.024	<100	-	-	-	[190]
Self-supported	40 vol. % Ni-BZCY (Ba(Zr_0.1_Ce_0.7_Y_0.2_)O_3-__δ_)	Uniaxially pressed	1000	900	4.2 × 10^−4^	-	-	-	-	[191]
Self-supported	40 vol. % Ni-BZCY (Ba(Zr_0.1_Ce_0.7_Y_0.2_)O_3-__δ_)	Uniaxially pressed	266	900	6 × 10^−3^	<100	0.5	2.17 × 10^−5^	-	[192]
BZCY	40 vol. % Ni-BZCY (Ba(Zr_0.1_Ce_0.7_Y_0.2_)O_3-__δ_)	Co-pressed	30	900	2.4 × 10^−3^	-	-	-	-	[193]
Self-supported	40 vol. % Ni-BCY (Ba(Ce_0.9_Y_0.1_)O_3-__δ_)	Cold isostatic pressing	230	800	5.88 × 10^−3^	-	-	-	-	[194]
40 vol. % Ni-BZCYYb	40 vol. % Ni-BZCYYb (Ba(Zr_0.1_Ce_0.7_Y_0.1_Yb_0.1_)O_3-__δ_)	Particle suspension coating	44	900	8.33 × 10^−3^	-	-	-	-	[195]
40 vol. % Ni-BCTb	40 vol. % Ni-BCTb (BaCe_0.95_Tb_0.05_O_3-__δ_)	Uniaxial pressing	90	850	0.068	-	-	-	-	[196]
α-Al_2_O_3_	60 vol. % Pd-YSZ	Paste-painting	18	900	0.387	<100	-	-	-	[197]
α-Al_2_O_3_	Pd/γ-Al_2_O_3_	Pore-filled	4–5	300	0.55	400	-	-	1000	[111]
α-Al_2_O_3_	Pd/YSZ-γ-Al_2_O_3_	Pore-filled	3.23	500	0.616	500	-	-	350	[117]
α-Al_2_O_3_	Pd/γ-Al_2_O_3_	CVD	10	300	~0.265	200	0.5	1.3 × 10^−3^	883 ^a^	[198]
α-Al_2_O_3_	Pd/γ-Al_2_O_3_	Pore-filled	10	300	~0.095	200	0.5	4.05 × 10^−4^	176 ^b^	[198]
α-Al_2_O_3_ (HF)	Pd-encapsulated (M-E)	Pore-filled	1.1	370	0.26	300	-	-	3800	[199]
α-Al_2_O_3_ (HF)	Pd-encapsulated nanopore (M-EN)	Pore-filled	1.6	370	0.25	300	-	-	8750	[199]
α-Al_2_O_3_ (HF)	Pd-nanopore (M-N)	Pore-filled + thin layer	2.6	370	0.13	300	-	-	550	[199]
α-Al_2_O_3_ (HF)	Pd-nanopore	Pore-filled + thin layer	4	400	0.56	50	-	-	6600	[200]
α-Al_2_O_3_	Pd/YSZ-γ-Al_2_O_3_	Pore-filled	~2	550	9.04 × 10^−3^	50	-	-	50	[201]
α-Al_2_O_3_	70% Pd-30% ZrO_2_	Pore-filled	~0.215	320	0.25	200	-	-	-	[202]
α-Al_2_O_3_	Pd/γ-Al_2_O_3_	Pore-filled	(8–10)	500		50	1	1.20 × 10^−6^	10,000	[203]

^a^ Recalculated value. N_2_ permeance 1.5 × 10^−9^ mol∙m^−2^∙s^−1^∙Pa^−1^ (Table 1 of reference). Hydrogen flux at 200 kPa of pressure difference: ~0.265 mol∙m^−2^∙s^−1^ (Figure 3a). Then hydrogen permeance is 1.32 × 10^−6^ mol∙m^−2^∙s^−1^∙Pa^−1^; ^b^ Recalculated value. N_2_ permeance: 2.7 × 10^−9^ mol∙m^−2^∙s^−1^∙Pa^−1^ (Table 1 of reference). Hydrogen flux at 200 kPa of pressure difference: ~0.095 mol∙m^−2^∙s^−1^. Then hydrogen permeance is 4.75 × 10^−7^ mol∙m^−2^∙s^−1^∙Pa^−1^
